# Highly branched  poly(β-amino ester) delivery of minicircle DNA for transfection of neurodegenerative disease related cells

**DOI:** 10.1038/s41467-019-11190-0

**Published:** 2019-07-24

**Authors:** Shuai Liu, Yongsheng Gao, Dezhong Zhou, Ming Zeng, Fatma Alshehri, Ben Newland, Jing Lyu, Jonathan O’Keeffe-Ahern, Udo Greiser, Tianying Guo, Fengzhi Zhang, Wenxin Wang

**Affiliations:** 1 Charles Institute of Dermatology, School of Medicine, University College Dublin, Belfield, 4 Dublin, Ireland; 2 Key Laboratory of Functional Polymer Materials, Ministry of Education, Institute of Polymer Chemistry, College of Chemistry, Nankai University, 300071 Tianjin, China; 3 School of Chemical Engineering and Technology, Xiʹan Jiaotong University, 710049 Xiʹan, China; 4 School of Pharmacy and Pharmaceutical Sciences, Cardiff University, CF103AT Cardiff, UK; 5 School of Pharmaceutical Science, Zhejiang University of Technology, 310014 Hangzhou, China; 6 Department of Polymer Science and Engineering, Zhejiang University, 310027 Hangzhou, China

**Keywords:** Biomaterials, Drug delivery, Polymer synthesis

## Abstract

Current therapies for most neurodegenerative disorders are only symptomatic in nature and do not change the course of the disease. Gene therapy plays an important role in disease modifying therapeutic strategies. Herein, we have designed and optimized a series of highly branched poly(β-amino ester)s (HPAEs) containing biodegradable disulfide units in the HPAE backbone (HPAESS) and guanidine moieties (HPAESG) at the extremities. The optimized polymers are used to deliver minicircle DNA to multipotent adipose derived stem cells (ADSCs) and astrocytes, and high transfection efficiency is achieved (77% in human ADSCs and 52% in primary astrocytes) whilst preserving over 90% cell viability. Furthermore, the top-performing candidate mediates high levels of nerve growth factor (NGF) secretion from astrocytes, causing neurite outgrowth from a model neuron cell line. This synergistic gene delivery system provides a viable method for highly efficient non-viral transfection of ADSCs and astrocytes.

## Abstract (web summary)

Therapeutics delivery by using non-viral vectors presents several challenges in the design of an appropriate polymeric structure. Here, the authors report a series of highly branched, biodegradable polymers which show high transfection efficiency in human multipotent adipose derived stem cells and primary astrocytes.

## Introduction

Cell and gene therapies have been considered for a range of neurological disorders^1,2^. Transplanted cells can either be used to secrete factors such as neurotrophic/neuroprotective agents, or they can be used to replace lost cell types^3,4^. Manipulating stem cells to further improve their beneficial secretome by overexpressing neuroprotrophic/neuroprotective growth factors prior to transplantation (ex vivo manipulation) is an attractive strategy for neural repair^5–7^. Alternatively, directly transfecting a major support cell (such as astrocytes) in vivo would allow secretion of factors to support damaged neurons^8^. One major hurdle for both strategies arises due to the fact that these cell types are typically hard to transfect.

Adipose-derived stem cells (ADSCs) are easy to isolate, expand and engraft, and can secrete a broad spectrum of angiogenic, neurotrophic factors and chemokines^9^. They are therefore an exciting cell resource for neural tissue regeneration with good clinical applicability^10,11^. ADSCs also have the ability to regulate the inflammatory response and hold antioxidant capacity^12^. Moreover, under the induction of specific genetic signals, ADSCs can differentiate into neuron-like or glia-like cells^13,14^. However, ADSCs are difficult to transfect^14^, thus driving the design of better gene delivery systems. Astrocytes are also hard to transfect showing far lower levels of transgene expression than other cell types^15,16^, making direct gene transfer into the brain somewhat limited^17^.

Gene transfection vectors, especially non-viral vectors have attracted increasing attention because of high safety profile, large gene loading capacity and ease of large-scale production^18–21^, but are consistently plagued by the unsatisfied gene transfection capability^22–24^. Lynn and Langer^25^ developed linear poly(β-amino ester)s (LPAEs) through the conjugation addition of amines to diacrylates. Owing to the broad monomer availability, the chemical composition of LPAEs is highly tailorable, and so far more than 2350 LPAEs have been developed^26–28^. The top-performing LPAE series have demonstrated their high gene transfection potency and biocompatibility on a variety of cell types^29–31^. In-depth mechanistic studies illustrate that the terminal units of LPAEs play a critical role in achieving high gene transfection efficiency^28,32^. For example, by altering the alcohol terminals to primary amines, the transfection efficiency can be improved by three orders-of-magnitude^33,34^.

As such, one can now envisage that dendritic poly(β-amino ester)s could be more conducive for efficient gene transfection, because, unlike LPAEs with only two terminals, dendritic poly(β-amino ester)s contain multiple terminal units. The three-dimensional (3D) topological structure of  dendritic poly(β-amino ester)s would provide a large chemical space for functionalization and would be more favorable for DNA condensation and protection from enzymatic degradation^35^. As such, recently we have developed highly branched poly(β-amino ester)s (HPAEs) as gene delivery vectors^36–39^. Both in vitro and in vivo studies demonstrated the high efficiency of HPAEs in gene transfection. Importantly, the inherent nature of highly branched structure: multiple terminal groups makes HPAEs a highly flexible and tailorable platform for the development of various functionality-enhanced gene delivery vectors aiming at different clinical targets.

Aside from the vector, the DNA cassette itself also plays a crucial role in determining overall gene transfection safety and efficiency. For instance, earlier results showed that by removing the prokaryotic sequences, minicircle DNA constructs (MC DNA), a supercoiled minimally sized DNA cassette, were capable of mediating a prolonged non-integrative transgene expression^40,41^.

**Fig. 1:**
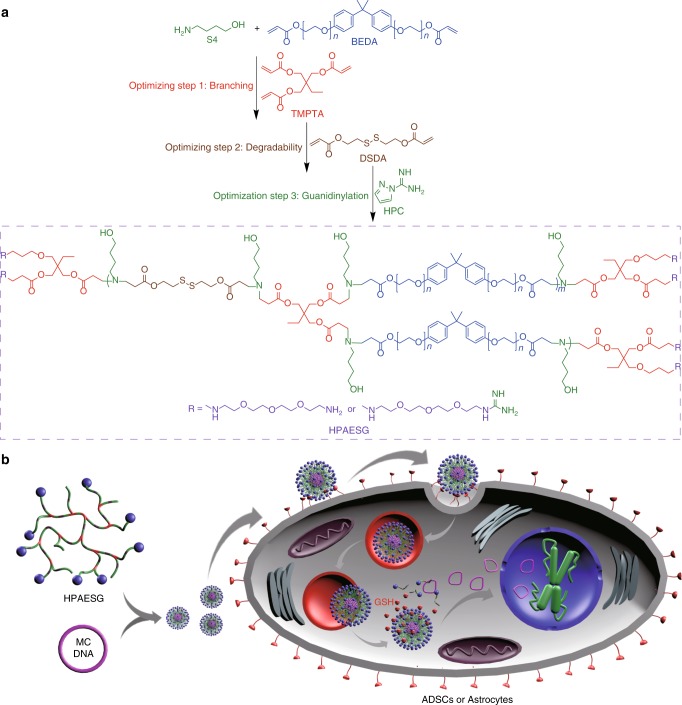
A synergistic gene delivery system for the transfection of ADSCs and astrocytes. **a** Iterative optimization of the branched structure, biodegradability, and guanidinylation identifies the leading HPAESG with high biocompatibility, enhanced cellular penetrating ability, and DNA release controllability. **b** The leading HPAESG condenses MC DNA to formulate nano-sized polyplexes, which can enter cells with high efficiency. After escaping from endo/lysosomes, the polyplexes are disassociated by glutathione (GSH)-mediated cleavage to release DNA, allowing safe and efficient gene transfection of ADSCs and astrocytes

The goal of this work is therefore to develop multifunctional HPAEs and combine them with a MC DNA for high-performance gene transfection of ADSCs and astrocytes. We aim to navigate the three key barriers associated with gene delivery: cytotoxicity, polyplex cellular uptake and DNA release. It is hypothesized that by integrating multiple disulfide bonds into HPAE backbone (HPAESS), glutathione-mediated biodegradation can be achieved. This will not only facilitate DNA release from HPAE/DNA polyplexes but also reduce the cytotoxicity of HPAEs after transfection. By adding guanidine groups to the terminals of HPAEs (HPAESG), the formation of multiple hydrogen bonds between guanidine moieties and polyhydroxyl compounds or heparan sulfate on the cell membrane will enhance cellular uptake of polyplexes. Three optimization steps are carried out (depicted in Fig. 1), with each being subjected to an iterative screening process, allowing assessment of the synergy between each modification. Optimization of the branched structure, disulfide bond content, and guanidinylation degree leads to a HPAESG, which, combining with MC DNA, gives a far superior transfection profile in both ADSCs and primary astrocytes.

## Results

### Identification of the optimal HPAEs for gene transfection

Numerous studies have demonstrated that for branched polymeric gene vectors, the different topological structures have substantial effects on DNA binding affinity and size, zeta potential and morphology of polyplexes, all of which are relevant to the ultimate transfection efficiency and cytotoxicity^36,42^. To better understand the structure-property relationship and determine the optimal branched structure for sequential functionalization, we began by synthesizing HPAEs with gradually transited structures. By utilizing 4-amino-1-butanol (S4) and bisphenol A ethoxylate diacrylate (BE) as functional monomers and varying the content of the branching monomer trimethylolpropane triacrylate (TMPTA) in the feed ratio, we prepared a total of five HPAEs and one LPAE with similar molecular weights (Supplementary Tables 1 and 2 and Supplementary Figs. 1 and 2). As the TMPTA feed ratio increases (Supplementary Table 2), the evolution of the molecular weight of the corresponding HPAE-based polymer becomes faster, as reflected by the gel permeation chromatography (GPC) curves (Supplementary Fig. 3), indicating the acceleration of oligomer combinations by increasing the branching unit amount. The attempt to calculate the branching degree (BD) from nuclear magnetic resonance (NMR) spectra (Supplementary Fig. 2) was failed due to the identical chemical environment of the three vinyl groups in TMPTA (Supplementary Fig. 4**)**. Instead, Mark-Houwink plots of the HPAEs were measured and the exponent (*α*) values were extracted to compare their extents of branching. As shown in Supplementary Table 2 and Supplementary Fig. 5, the *α *value for HPAE-1 to HPAE-5 was 0.48, 0.46, 0.44, 0.37, and 0.32, respectively, which was less than that of LPAE: 0.63, demonstrating the dendritic nature of HPAEs. Moreover, smaller *α* values were obtained from those HPAEs with higher TMPTA to BE feed ratios, corresponding to more spherical morphologies and thereby higher branching degrees. It is noteworthy that, although those HPAEs have broad molecular weight distributions with dispersity (*Đ*) values ranging from 2.94 to 6.80, which is a common feature for polymers from polycondesation reations^43,44^, their branched structures and molecular weights are consistent from batch to batch as shown in Supplementary Fig. 6 and Supplementary Table 3, highlighting their potential for the clinical applications.

**Fig. 2:**
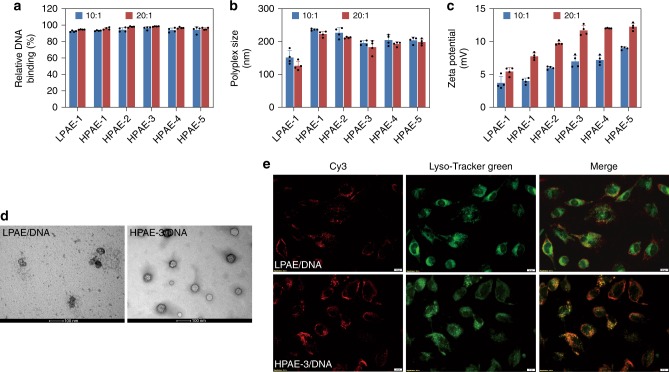
Biophysical characterization of HPAEs differing in branched structure. **a** All the polymers have strong and similar DNA binding affinity. **b** All the polyplexes have sizes < 250 nm and LPAE/DNA polyplexes have a smaller size in comparison with HPAE/DNA counterparts. **c** As the branching degree increases, the zeta potential of polyplexes increases and reaches at a stable level at 12 mV. **d** LPAE/DNA and HPAE-3/DNA polyplexes manifest substantially different morphologies, the former is spherical while the latter is toroidal, the scale bars represent 100 nm. **e** HPAE-3/DNA polyplexes show much stronger cellular uptake and endo/lysosomal escape efficiency, compared to the LPAE/DNA counterparts. The scale bars represent 20 μm. Data are shown as average ± SD; *n* = 4

Next, we characterized and compared three physiological properties of the polymer/DNA polyplexes: binding efficiency, size and surface charge. Picogreen assays showed that at polymer/DNA weight ratios (*w*/*w*) of 10:1 and 20:1, all polymers showed a strong DNA binding affinity (Fig. 2a). Dynamic light scattering (DLS) measurements further showed that all the polyplexes had sizes less than 250 nm (Fig. 2b). Of all the HPAE/DNA polyplexes, HPAE-3/DNA were slightly smaller than the other HPAE counterparts. The stability of LPAE/DNA and HPAE-3/DNA polyplexes (*w*/*w* = 20:1) was also tested. As shown in Supplementary Fig. 7, HPAE-3/DNA polyplexes remained stable in the presence of bovine serum albumin (BSA). Whereas, sizes of LPAE/DNA polyplexes increased dramatically, which is correlated to a recent observation by Anderson and colleagues^45^. With respect to the surface charge, all the polyplexes showed moderate positive zeta potentials (Fig. 2c). We speculate that the deviation in polyplex size and zeta potential between HPAEs and LPAE is possibly due to the different interaction mechanisms of polymers with DNA. As the branching degree increases, the polymers gradually transit from a linear, flexible structure to a globular, more rigid structure, meanwhile the number of terminal primary amines per polymer molecule also increases, contributing to the rise in positive charge. Since the DNA chain is highly charged and rigid because of the electrostatic repulsion, and HPAEs are globular, they perhaps condense DNA into a toroidal nano-ring structure, as opposed to LPAEs condensing the DNA into a more compact/spherical nanoparticle of smaller size. To validate our speculation, we used transmission electron microscopy (TEM) to observe the morphologies of different polyplexes. As shown in Fig. 2d, the HPAE-3/DNA polyplexes manifested a uniform and toroidal morphology while the LPAE/DNA counterparts showed a spherical morphology. We believe that the different size, zeta potential as well as morphologies would lead to different cellular uptake efficiency of polyplexes. Using Cy3 (a red fluorescence dye, used for DNA labeling) and Lyso-Tracker Green (a green fluorescence indicator of endo/lysosomes) as reporters, we showed that on HeLa cells, HPAE/DNA polyplexes indeed have a higher cellular uptake efficiency than the LPAE/DNA counterparts (Fig. 2e). The endo/lysosomal escape ability of the polyplexes was also compared by quantifying the Person correlation coefficient (PCC) using Image J. As shown in Supplementary Fig. 8, at the *w*/*w* ratio of 20:1, the average PCC of the HPAE-3/DNA and LPAE/DNA polyplexes calculated from three representative cell images were 0.30 and 0.62, respectively. The much lower PCC indicates that the HPAE-3/DNA polyplexes exhibit a stronger endo/lysosomal escape ability in comparison with the LPAE/DNA counterparts.

**Fig. 3:**
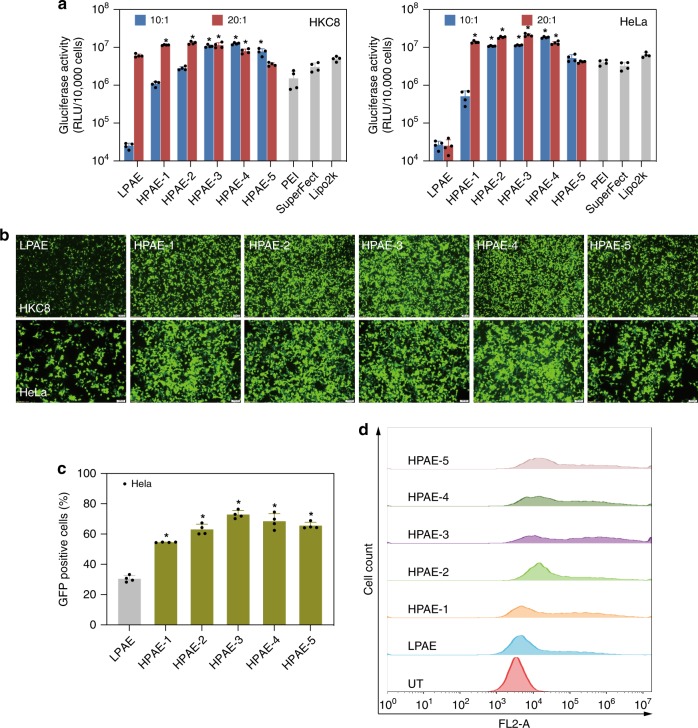
HPAEs show much higher transfection efficacy than LPAE and commercial reagents. **a** Gluciferase activity of HKC8 and HeLa cells after 48 h transfection with different vectors at *w*/*w* ratios of 10:1 and 20:1. Data points marked with asterisks (*) are statistically significantly higher relative to the Lipo2k group. **b** Fluorescence images of HKC8 and HeLa cells 48 h post transfection with various vectors at the *w*/*w* ratio of 20:1. The scale bars for HKC8 cells represent 200 μm, for HeLa cells represent 100 μm. **c** The percentage of GFP-positive Hela cells after transfection, asterisk indicates significantly higher transfection efficiency in comparison with the LPAE group. **d** Representative histogram distributions of untreated (UT) HeLa cells and the ones after transfection with HPAEs at the *w*/*w* = 20:1. Data are shown as average ± SD; *n* = 4

The gene transfection efficiency of the HPAEs was evaluated on HKC8 and HeLa cells, with three widely used commercial transfection reagents polyethylenimine (PEI), SuperFect and Lipofectamine 2000 (Lipo2k) as positive controls. As shown in Fig. 3a, Gluciferase activity of cells after transfection with HPAEs was substantially higher than that of the LPAE. Moreover, at the *w*/*w* ratio of 20:1, all HPAEs outperformed the three commercial reagents and orders-of-magnitude higher Gluciferase activity was observed. The images of cells after transfection with green fluorescent protein (GFP) further validated the high-transfection capability of the HPAE library (Fig. 3b–d and Supplementary Figs. 9 and 10). Our results conclude that the branched structure is much more conducive for gene transfection, with a relatively moderate branching degree (HPAE-3) showing higher transfection capability than the more branched HPAE-4 and HPAE-5 or less branched HPAE-1 and HPAE-2. This trend is in agreement with our previous report^36^, and we speculate that this is probably due to a good balance between polymer chain flexibility and rigidity. Moreover, HPAEs did not induce obvious cytotoxicity (Supplementary Fig. 11). To explore the possible mechanism of the good biocompatibility of HPAEs, the degradation of HPAE-3 was studied (Supplementary Methods). As shown in Supplementary Fig. 12, after 6 h of incubation in phosphate buffer solution (PBS), the molecular weights of HPAE-3 and LPAE both decreased obviously. The cytotoxicity of the degraded byproducts of HPAE-3 was further evaluated. They exerted negligible cytotoxicity; even if the concentration of degraded byproducts was up to 250 μg mL^−1^, over 90% of cell viability was maintained (Supplementary Fig. 13). All these data confirm that HPAEs are biodegradable and that the degraded byproducts are highly biocompatible without inducing any obvious cytotoxicity during the gene transfection process.

### Integration of disulfide bonds to improve biodegradability

Vector degradability matters for gene transfection by affecting vector cytotoxicity and DNA release^18,42,46^. An intrinsic feature that distinguishes poly(β-amino ester)s from other vectors is its hydrolytic degradability in the physiological environment. However, the hydrolysis process is random and the duration ranges from several hours to a few days, which would result in poorly timed or off-target DNA release^47,48^, therefore spatiotemporally controlled DNA release is highly desired for gene transfection. GSH is a natural tripeptide with a concentration inside cells of around 2–10 mM while outside it is only 1–10 μM^18,49^. Cationic polymers containing disulfide bonds in the backbone can maintain extracellular stability, but can be cleaved quickly upon cell entry due to the high concentration of GSH causing the thiol-disulfide interchange reaction. Numerous researches have incorporated disulfide bonds into cationic polymers to induce material degradation and ultimate high-transfection efficacy^50–53^. Therefore, the integration of disulfide bonds into the backbone of HPAEs is of great significance for two reasons. Firstly, the branched structure has demonstrated to have very strong DNA condensation affinity (Fig. 2a), which would potentially lead to slow DNA release by hydrolysis. Secondly, the high-charge density (Fig. 2c) derived from the multiple terminal primary amines could induce cytotoxicity. To realize this realm, disulfanediylbis(ethane-2,1-diyl) diacrylate (DSDA) was introduced to the monomer combination for HPAE-3 synthesis (Supplementary Table 4 and Supplementary Fig. 14), and five disulfide-containing HPAEs (HPAESSs) with different DSDA feed ratios were produced via Michael addition approach (Supplementary Table 5 and Supplementary Figs. 15 and 16).

**Fig. 4:**
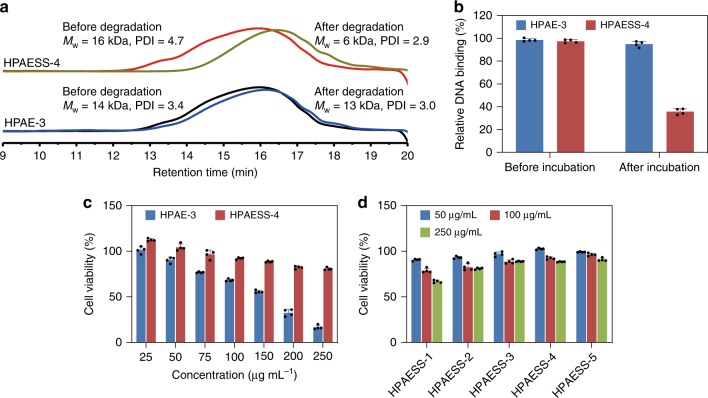
Enhanced biodegradability benefits DNA release and low cytotoxicity. **a** GPC traces of HPAE-3 and HPAESS-4 before and after incubation with 10 mM GSH show that the HPAESS-4 can be degraded quickly by GSH. **b** The relative DNA binding affinity of HPAESS-4 decreased significantly after incubation with 10 mM GSH, indicating its enhanced degradability by the integrated disulfide bonds in the backbone. **c** At a series of polymer concentrations, cell viability of primary astrocytes incubated with HPAESS-4 was much higher than that with HPAE-3. **d** At a series of polymer concentrations, cell viability of primary astrocytes increased with the content of disulfide bonds in polymer backbones. Data are shown as average ± SD; *n* = 4

**Fig. 5:**
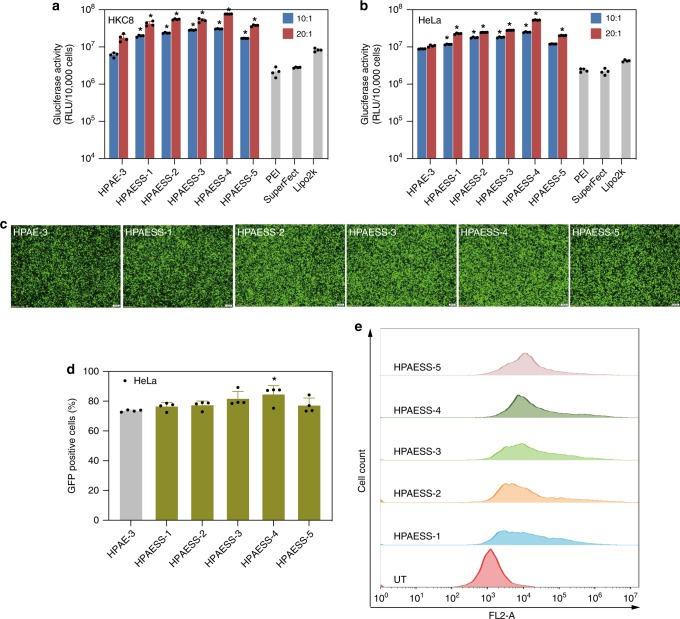
Disulfide-containing HPAESSs show higher transfection efficacy than HPAE-3 and commercial reagents. **a**, **b** Gluciferase activity of HKC8 and HeLa cells after transfection with different vectors. Data points marked with asterisks (*) are statistically significantly higher relative to the HPAE-3 group. **c** Fluorescence images of HeLa cells 48 h post transfection with various vectors at the *w*/*w* ratio of 20:1. The scale bars represent 200 μm. **d** The percentage of GFP-positive cells after transfection, asterisk indicates significantly higher transfection efficiency in comparison with the HPAE-3 group. **e** Representative histogram distributions of UT HeLa cells and the ones after transfection with HPAESSs at the *w*/*w* = 20:1. Data are shown as average ± SD; *n* = 4

We firstly used GPC and Picogreen assays to confirm whether HPAESSs can be cleaved by GSH to enhance DNA release. The molecular weights of HPAESS-4 before and after incubation in 10 mM GSH solution for 1 h were shown in Fig. 4a. It is evident that after incubation with GSH, the molecular weight of HPAESS-4 substantially decreased from 16 to 6 kDa. In contrast, the molecular weight of HPAE-3, a polymer without disulfide bonds in the backbone, only decreased slightly from 14 to 13 kDa. In agreement with the GPC results, Picogreen assays further showed that in the presence of GSH, the DNA binding affinity of HPAESS-4 was significantly dropped from 97 to 35%, in sharp contrast with that of HPAE-3 (Fig. 4b). Moreover, at high concentrations ( > 100 μg mL^−1^), HPAESS-4 preserved substantially higher viability of primary astrocytes (Fig. 4c). Further, as the content of disulfide bonds increased, HPAESSs showed decreasing cytotoxicity (Fig. 4d). Similar to HPAEs, all the HPAESSs could effectively condense DNA to form nano-sized polyplexes with moderate surface charge (Supplementary Fig. 17). As a consequence, as confirmed by the Gluciferase activity analysis and GFP measurements, the gene transfection efficiency of HPAESSs is enhanced to a different degree by the introduction of disulfide bonds and HPAESS-4 exhibits the highest efficiency (Fig. 5). The fact that both enhanced DNA release and reduced cytotoxicity can be achieved simultaneously makes this strategy advantageous for the development of HPAE-based gene delivery vectors, in terms of ease of integration and improved performance.

### Guanidinylation to facilitate polyplex cellular uptake

Cellular uptake of polyplexes poses another key barrier for achieving high gene transfection efficiency. In 2000, breakthrough studies by Wender et al.^54^ discovered that arginine-rich cellular penetrating peptides (CPPs) can form multiple hydrogen bonds with the phosphate, carboxylate, or sulfate groups located on cell surfaces, facilitating greater transportation of cargos (e.g., drug, DNA, siRNA, protein, quantum dots etc.) across the cellular membrane. Inspired by CPPs, a variety of non-viral gene vectors bearing guanidine moieties have been developed^55,56^. The multivalence of guanidine moieties can enhance polyplexes cellular membrane affinity and improve the cellular uptake efficiency of polyplexes.

**Fig. 6:**
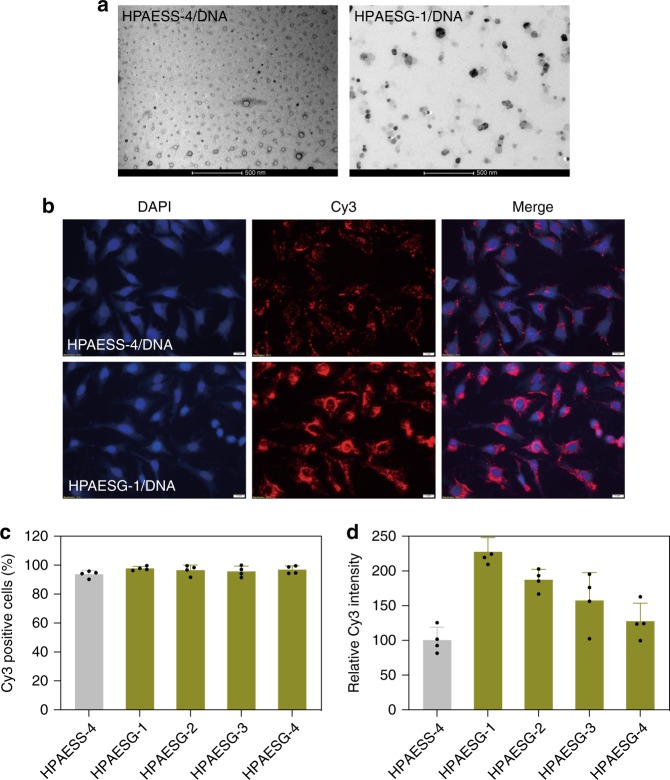
Improvement of polyplex cellular uptake by guanidinylation of HPAESSs. **a** TEM images show that HPAESS-4/DNA and HPAESG-1/DNA polyplexes have different morphologies, the former is uniform and toroidal while the latter is polyhedral, the scale bars present 500 nm. **b** Fluorescence images of HeLa cells after incubation with HPAESS-4/DNA and HPAESG-1/DNA polyplexes for 4 h at the *w*/*w* ratio of 20:1, the latter clearly show higher cellular uptake efficiency, the scale bars present 20 μm. **c** The cellular uptake efficiency of different HPAESG/DNA polyplexes showing similar percentages of Cy3-positive HeLa cells. **d** Cells after treatment with HPAESG/DNA polyplexes have higher relative fluorescence intensity than the HPAESS/DNA counterparts. Data are shown as average ± SD; *n* = 4

**Fig. 7:**
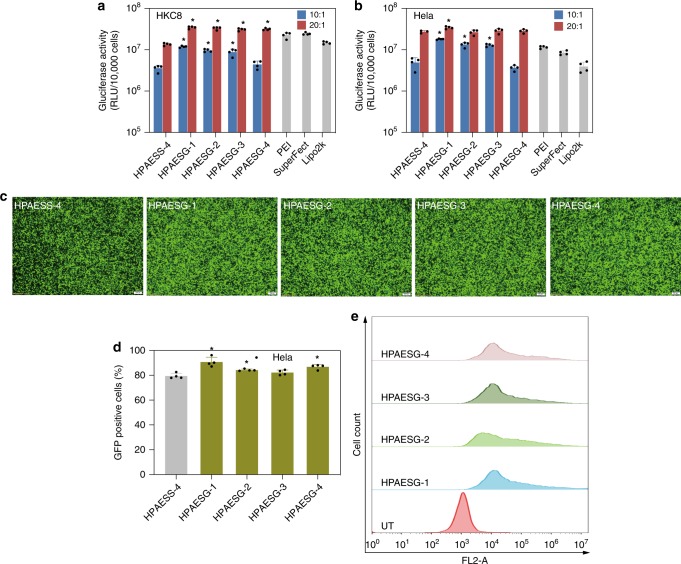
The functionalization of HPAESS-4 with guanidine further improves transfection capability. **a**, **b** Gluciferase activity of HKC8 and HeLa cells after transfection with different vectors. Data points marked with asterisks (*) are statistically significantly higher relative to the HPAESS-4 group. **c** Fluorescence images of HeLa cells 48 h post transfection with various vectors at *w*/*w* ratios of 20:1. The scale bars represent 200 μm. **d** The percentage of GFP-positive cells after transfection, asterisk indicates significantly higher transfection efficiency in comparison with the HPAESS-4 group. **e** Representative histogram distributions of UT HeLa cells and the ones after transfection with HPAESGs at the *w*/*w* = 20:1. Data are shown as average ± SD; *n* = 4

Given that cellular uptake of polyplexes substantially affects the overall gene transfection efficiency of poly(β-amino ester)-based gene delivery systems, and the fact that HPAEs offer multiple reaction sites for further functionalization, one can envisage that guanidine groups can be incorporated to the peripheries of HPAE to enhance the cellular uptake of polyplexes. To this end, HPAESS-4 was selected for guanidinylation at a 10%, 30%, 60 and 100% degree (Supplementary Figs. 18 and 19, denoted as HPAESG-1 to HPAESG-4, respectively). The gunidinylation did not reduce the positive charges of HPAESS, instead, the formulated HPAESG/DNA polyplexes exhibited a slightly bigger size and comparatively higher zeta potential compared with the HPAESS/DNA counterparts (Supplementary Fig. 20), which is in accordance with previous studies^55^. Interestingly, some of the HPAESG-1/DNA polyplexes manifested a polyhedral morphology in comparison with that of the HPAESS/DNA counterparts exhibiting a uniform toroidal morphology (Fig. 6a). The effects of guanidinylation on promoting polyplex cellular uptake were evaluated on HeLa cells using Cy3 as a fluorescence reporter. After 4 h of incubation, there were numerous HPAESG-1/Cy3-DNA polyplexes inside the cells and the majority were accumulating around the nucleus (Fig. 6b). In contrast, the HPAESS-4/Cy3-DNA polyplexes showed a lower uptake efficiency. Furthermore, although the percentage of Cy3-positive cells after treatment with different polyplexes was similar (Fig. 6c), HPAESG/DNA polyplexes mediated much stronger fluorescence than the HPAESS-4/DNA counterparts (Fig. 6d). In contrast to the previous report^55^, here as the guanidinylation degree increased, the cellular uptake efficiency of HPAESG/DNA polyplexes decreased. Correspondingly, Gluciferase activity of cells after transfection with HPAESG library also gradually decreased with HPAESG-1 showing the highest transfection efficiency (Fig. 7). These results demonstrate that guanidinylation can indeed enhance the affinity of HPAESG/DNA polyplexes with cells, however, it is not simply a case of more guanidinylation giving better transfection.

### HPAESG-1 shows high efficacy in ADSCs and astrocytes

We next transfected rADSC, hADSC, Neu7 astrocytes, and primary astrocytes using optimized HPAESG-1. As shown in Supplementary Fig. 21, HPAESG-1 exhibited high gene transfection activity on all the cell types. An approximate 1–4 orders of magnitude increase in transfection was shown compared to LPAE, which was also 15–106-fold higher than that of PEI, SuperFect and Lipo2k. For both hADSC and primary astrocytes, the Gluciferase activity after transfection by HPAESG-1 was still 15–18-fold stronger than the Lipo2k. Such high gene transfection performance by HPAESG-1 is outperformed most of the reported polymer-based vectors. For instance, a previous study using a cyclized poly(2-(dimethylami- noethyl) methacrylate) (PDMAEMA) to transfect hADSCs and Neu7, showing that Gluciferase activity of hADSC after transfection was only similar or even slightly lower than that mediated by SuperFect and PEI^57^. In another study, Gluciferase activity of Neu7 after transfection with a bio-reducible DSP8 polymer at its optimal *w*/*w* ratio of 3:1 was slightly higher than that mediated by SuperFect^16^. In contrast, here, the Gluciferase activity of ADSCs and astrocytes after transfection with HPAESG-1 is orders-of-magnitude higher than that mediated by PEI, SuperFect and Lipo2k. The high gene transfection capability of HPAESG-1 in ADSC and astrocytes was further compared with that of FuGENE, Xfect and Lipofectamine 3000 (Lipo3000). As shown in Supplementary Fig. 22, Gluciferase activity of rADSC and astrocytes after transfection with HPAESG-1 at the *w*/*w* ratio of 20:1 was between 4.6 and 170-fold higher than that mediated by FuGENE, Xfect and Lipo3000. Altogether, these results demonstrate the high gene transfection capability of HPAESG-1 in neurodegenerative disease relevant cells. Furthermore, HPAESG-1 is well tolerated by all cell types at the effective dosage, with at least 90% cell viability being preserved, starkly contrasting the severe cytotoxicity induced by PEI, SuperFect and Lipo2k (Supplementary Fig. 23).

### HPAESG-1/MC DNA shows high efficacy in ADSCs and astrocytes

**Fig. 8:**
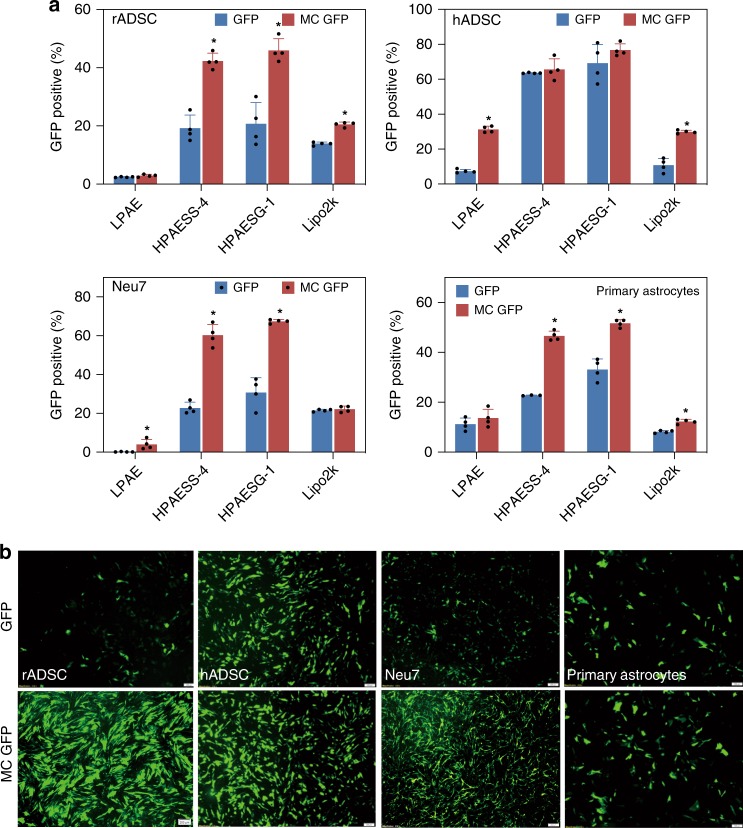
MC DNA is superior to standard DNA in transfection of ADSCs and astrocytes. **a**, **b** HPAESG-1/MC GFP exhibits higher gene transfection efficiency than HPAESG-1/GFP at the *w*/*w* ratio of 20:1. Data are shown as average ± SD; *n* = 4. Data points marked with asterisks (*) are statistically significant relative to the standard GFP group. The scale bars represent 200 μm

To further improve the transfection performance of HPAESG-1, we optimized the DNA itself by constructing a MC DNA encoding for GFP (MC GFP, 3.0 kbp). Interestingly, the difference in plasmid size significantly affects the physiological characteristics of the formulated polyplexes. All the polyplexes formulated from MC DNA showed an obviously smaller size in comparison with the counterparts formulated from normally sized 5.6 kbp GFP DNA (Supplementary Fig. 24). Across all the cell types, MC GFP mediated substantially higher transfection efficiency than the standard GFP regardless of gene vectors used; HPAESS-4 and HPAESG-1 showed significantly higher transfection efficiency than the LPAE and Lipo2k, however, HPAESG-1/MC GFP exhibited the most robust gene transfection efficiency: in rADSC, hADSC, Neu7, and primary astrocytes, the efficiency was 46%, 77%, 67%, and 52%, respectively (Fig. 8). These are very promising results because previously, the highest transfection efficiency of hADSCs achieved by Yang et al.^58^ was only 24%. Similarly, previous multiple repeats of lipofection only achieved 19% transfection efficiency in primary astrocytes^59^. These results show that HPAESG-1 in combination of MC DNA is capable of enhancing the gene transfection efficiency of ADSCs and astrocytes significantly. Moreover, HPAESG-1/MC GFP show high biocompatibility, with over 90% of cell viability being maintained (Supplementary Fig. 25).

### High levels of nerve growth factor expression in astrocytes

**Fig. 9:**
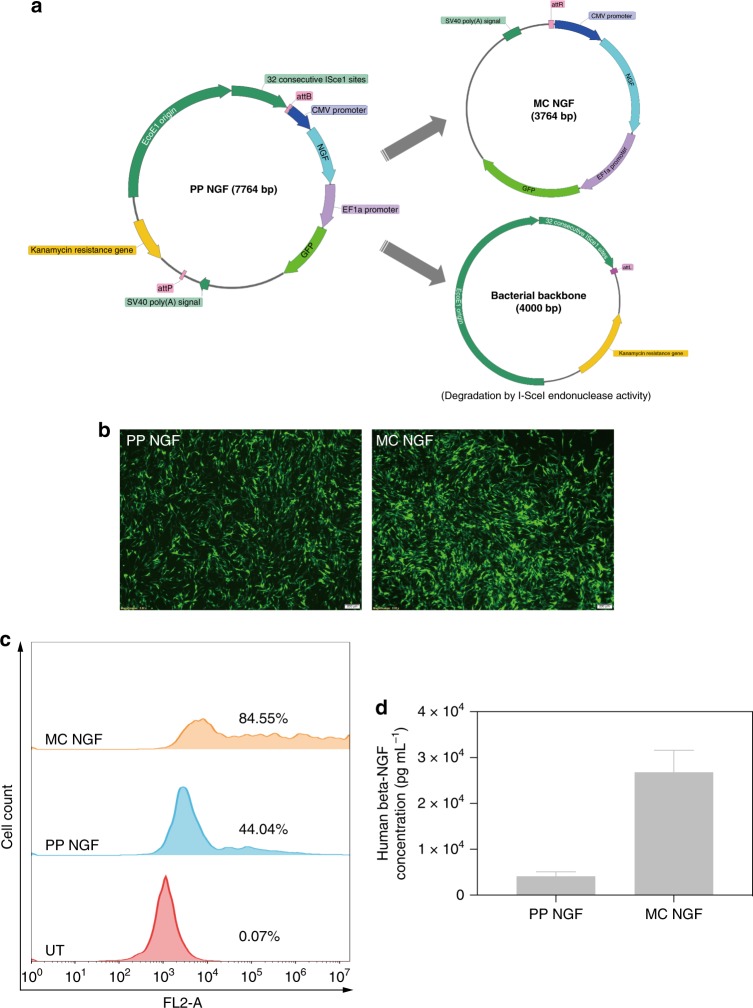
Construction of NGF encoding plasmids and evaluation of transfection efficacy. **a** Production of MC NGF in ZYCY10P3S2T *E. coli*. MC are produced from Parental plasmid using PhiC31 Integrase system. **b** GFP expression of Neu7 after transfection with HPAESG-1/PP NGF and HPAESG-1/MC NGF at the *w*/*w* = 20:1. The scale bars represent 200 μm. **c** Representative histogram distributions of UT Neu7 and the ones after transfection with HPAESG-1/PP NGF and HPAESG-1/MC NGF at the *w*/*w* = 20:1. **d** Human beta-NGF concentration of supernatants from Neu7 astrocytes after transfection with HPAESG-1/PP NGF or HPAESG-1/MC NGF at the *w*/*w* = 20:1. Data are shown as average ± SD; *n* = 2

**Fig. 10:**
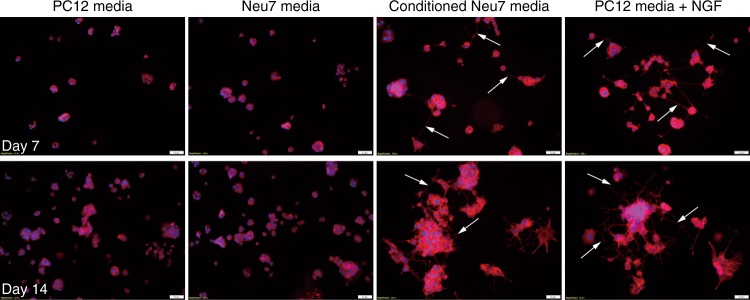
Fluorescent images of PC12 after incubation with different media. Arrows indicate the outgrowth of neurite. The cell nucleus were stained with DAPI (blue), F-actin was stained with Alexa Fluor 594 (red). The scale bars represent 50 μm

The intended application target of the HPAESG-1/MC DNA polyplexes is neurodegenerative diseases, and thus a therapeutic gene—nerve growth factor (NGF) encoding plasmid PP NGF (7.7 kbp) and its MC construct MC NGF (3.7 kbp) were further constructed and used for transfection in Neu7 and primary astrocytes. NGF plays a critical protective role in the development and survival of sympathetic, sensory and forebrain cholinergic neurons by promoting neurite outgrowth and nerve cell recovery^60–62^. Given the strong dependency of the neurons on NGF, effective delivery of NGF encoding plasmid to relevant cells to promote NGF secretion is a promising strategy for gene therapy of neurodegenerative diseases^63–65^. The PP NGF was synthesized by subcloning the NGF fragment into the parental plasmid MN511A1 (PPMN511A1, 7063 bp) (Supplementary Figs. 26 and 27). MC NGF was then produced in ZYCY10P3S2T E. coli using the PhiC31 Integrase system (Fig. 9a and Supplementary Fig. 28). There was a GFP tag in the plasmids, therefore, both the GFP and NGF expression could be quantified with flow cytometry and ELISA (Supplementary Fig. 29), respectively. The most favorable parameters for a high level of human beta-NGF expression was first identified and it was found that in general, a relatively higher cell density, DNA dosage, longer transfection time, and serum-free condition were more favorable for NGF accumulation in the cell supernatant while no obvious cytotoxicity was induced. Figure 9 and Supplementary Fig. 30 showed that at the *w*/*w* = 20:1, the GFP expression in Neu7 and primary astrocyte transfected by HPAESG-1/MC NGF was up to 84.55% and 59.80%, respectively, even higher than the HPAESG-1/ MC GFP counterpart as reported above, which is likely due to the serum-free condition used. In contrast, only 44.04% and 31.23% GFP expression was achieved by the PP NGF. The NGF expression in the supernatants of Neu7 and primary astrocytes after transfection with the HPAESG-1/ MC NGF was measured to be at high levels as well, which was 6.5- and 12.3-fold higher than that mediated by the HPAESG-1/PP NGF counterparts (Fig. 9d and Supplementary Fig. 30c). These results demonstrate not only the superiority of MC DNA in gene transfection compared to normally sized plasmids, but also the robust transfection capability of the HPAESG-1/MC DNA polyplexes in delivering and mediating the expression of therapeutic genes in neurodegenerative disease relevant cells, highlighting its great applicability for potential clinical gene therapy. Bioactivity of the secreted NGF was further assessed by exploring its ability to promote neurite outgrowth in pheochromocytoma cells (PC12), and thus conditioned cell culture experiments were carried out. PC12 cells were incubated under four different conditions: standard PC12 cell culture media (DMEM with high a glucose concentration, PC12 media group), standard Neu7 cell culture media (DMEM with a low glucose concentration, Neu7 media group), conditioned Neu7 cell culture media (media saved from Neu7 cells after transfection with HPAESG-1/MC NFG, the concentration of NGF was determined to be 26.9 ng mL^−1^, Conditioned Neu7 media group) and standard PC12 cell culture media but with 50 ng mL^−1^ recombinant NGF (PC12 + NGF group), respectively. As shown in Fig. 10, after 7 days, the PC12 media group and Neu7 media group did not show any neurite outgrowth (as expected when NGF is absent). In contrast, clearly visible neurite outgrowth was detected in the conditioned Neu7 media group and the PC12 + NGF group. Owing to the higher concentration of NGF, the PC12 + NGF media promoted the outgrowth of neurite more significantly, as evidenced by the higher number and longer neurites. After 14 days of incubation, similar to the PC12 + NGF media, much more obvious neurite outgrowth was observed in the conditioned Neu7 media group. However, although the cell density increased substantially in the PC12 media group and Neu7 media group, there was still no neurite outgrowth detected. All these results demonstrate the high bioactivity of the NGF produced by the astrocytes, which is secreted to the surrounding environment. We also tried to directly transfect the PC12 with the HPAESG-1/MC NGF polyplexes. At the *w*/*w* ratio of 20:1, although around 18.76% GFP expression was observed, unfortunately, very limited neurite outgrowth was detected, as shown in Supplementary Fig. 31. This is because the level of NGF expression in PC12 cells was too low as confirmed by ELISA (< 5.0 pg mL^−1^).

## Discussion

If ex vivo or in vivo cell manipulation is to reach the clinical use, the transfection efficiency and the safety of non-viral vectors have to be drastically improved. Some of the most successful developments in the non-viral field have been through the development of poly(β-amino ester)s^27,66^. To date, most of this type of polymers has been focused upon linear structures^14,25^. However, we have recently shown that HPAEs may have a substantial advantage over their linear counterpart^36,37^. Herein, we have performed a stepwise modification and optimization strategy to break the hurdles of poor non-viral gene vectors.

In this work, we focused on two specific cell types relevant to gene therapy strategies for neurodegenerative disorders: ADSCs and astrocytes. Apart from the clinical implications of being able to transfect these cells, they were also chosen as a tough test bed for the development of an efficient vector. Using HKC8 and HeLa cells for the optimization steps, an iterative modification strategy was employed to optimize HPAEs to navigate the key barriers of gene transfection, including cytotoxicity, polyplex uptake, and DNA release. We started with optimization of the branched structure by synthesizing five HPAEs differing in branching degree. Polyplex characterization and gene transfection indicate that HPAEs with a moderate branching degree is more favorable for transfection. Increasing amounts of disulfide bonds were introduced into the backbone of HPAE-3, our results showed that the degradability of HPAESS was enhanced, with a corresponding increase in the rate of DNA release, and the cytotoxicity was significantly reduced. This work therefore corroborates that shown with PEI by Breunig et al.^67^. HPAESS-4 was further modified by guanidine for improved cellular penetrating ability. Interestingly, the lower guanidinylation degree is more effective, and at a high *w*/*w* ratio (e.g., 20:1) all the HPAESGs have similar transfection capability.

Turning towards the cell types of interest, the leading polymer from the third optimization step (HPAESG-1) was initially evaluated using a standard DNA construct. Up to 106-fold higher transfection efficiency was achieved by HPAESG-1 in comparison with the commercial reagents, whilst over 90% cell viability was preserved. Having optimized the vector, it was then desired to analyze whether a small DNA construct could add another layer of synergistic improvement. The accessory sequences for propagation of plasmid DNA can have adverse effects such as the viral/bacterial sequence triggering immune responses, or the antibiotic resistance sequences potentially transferring to the host^68^. Additionally, high level and long-term expression of functional proteins is highly sought after for gene therapy, to avoid high cost and repeated gene administrations. To this end, MC DNA constructs have been designed by removing the prokaryotic sequences resulting in a supercoiled minimally sized DNA cassette^40,41,69^.

When MC DNA was combined with HPAESG-1, high-transfection efficacy in rADSC, hADSC, Neu7 and primary astrocytes was achieved, indicating the synergistic effects of vector structure, degradability, functionalization and DNA construct. To demonstrate the potential clinical applicability, a therapeutic MC NGF was further constructed and it was found that high level of NGF expression in astrocytes was achieved. Importantly, the NGF showed high bioactivity to promote neurite outgrowth from PC12 cells. All these results demonstrate that the synergistic gene delivery system comprising an optimized gene vector and an optimal DNA construct can achieve robust transfection activity and high safety simultaneously, implying the great potential of this system in the treatment of neurological diseases. In addition, given the ease of operation and versatility, our synergistic modification strategy may be used for the development of various non-viral gene delivery vectors targeting diverse cell types and diseases.

## Methods

### Iterative modification and optimization of HPAEs

As outlined in Supplementary Table 1, to synthesize HPAE-1 to HPAE-5, the stoichiometric ratio of the overall amines to acrylates was kept at 1:1.2. Monomers S4, TMPTA and BE with varying ratios were dissolved in dimethyl sulfoxide (DMSO), respectively, to an overall monomer concentration of 500 mg mL^−1^. The reaction occurred at 90 ^o^C to generate acrylate terminated base polymers. Afterwards, DMSO was added to dilute the reaction mixture to 100 mg mL^−1^, and then a second amine DT was added to end-cap the generated base polymers at room temperature for 48 h. To purify the polymers, the reaction mixtures were precipitated into diethyl ether three times. Polymers were collected, dried under vacuum for 24 h at room temperature and then stored at −20 ^o^C for the following studies. The corresponding linear polymer LPAE, was prepared following the same procedures using bulk polymerization without triacrylate monomer TMPTA or the solvent DMSO.

The HPAE-3 was functionalized by introducing disulfide bonds to the backbone. As outlined in Supplementary Table 4, the stoichiometric ratio of the overall amines to acrylates was 1:1.2, TMPTA and S4 and the monomer feed ratios were the same as above, while varying amounts of DSDA and BE were employed. Five HPAEs that differed in the content of disulfide component, termed as HPAESS-1 to HPAESS-5, respectively, were prepared as above. The HPAESS-4 was further functionalized by introducing multiple guanidine groups at the periphery primary amines (calculated to be 0.64 mmol g^−1^). Briefly, 94 mg HPAESS-4 was dissolved in DMSO, and then the guanidinylation agents 1H-pyrazole-1-carboxamidine hydrochloride (HPCH) and N,N-diisopropylethylamine (DIPEA) (in equivalent mole, 0.006, 0.018, 0.036, and 0.06 mmol, respectively) were added. After 2 days of reaction at room temperature, the reaction mixtures were directly dialyzed against DMSO for 3 days. The final products, HPAESG-1 to HPAESG-4 of 10%, 30%, 60% or 100% guanidinylation degree, respectively, were lyophilized.

### HPAESS degradation in the presence of GSH

Fifty milligram of polymers were dissolved in 1 mL sodium acetate buffer, followed by addition of GSH solution (20 mM, 1 mL). The mixtures were kept stirring at room temperature (RT) for 1 h and then lyophilized immediately. Molecular weights of the degraded products were measured with GPC.

### Polyplex size and zeta potential

To measure the size and zeta potential, polyplexes were firstly prepared by adding polymer solution into DNA solution at a 1:1 volume ratio, mixing and incubating for 10 min, 2 μg Gluc DNA was used for each sample. The polyplexes were then diluted to 1 mL with PBS. Size and zeta potential were measured with DLS and electrophoretic light scattering, respectively. To test polyplex stability in the presence of protein, LPAE/DNA and HPAE-3/DNA polyplexes (*w*/*w* = 20 :1) were prepared as above and then diluted to 1 mL with BSA containing deionized water, the final concentration of BSA was 0.5%. Size of the polyplexes was measured with DLS immediately at 0, 0.5 and 1 h after preparation, respectively.

### Subcloning of NGF into PPMN511A1

The parental plasmid (PPMN511A1) was used as a minicircle construction system (System Biosciences). The PPMN511A1 (vector, V) (7.0 kbp) was digested by *Xba*I and *Bam*HI enzymes. Simultaneously, *Xba*I and *Bam*HI enzymes digestion sites were used to cut the human NGF gene fragment (insert, I) (0.7 kbp) from pEX-A2-NGF plasmid, which was synthesized by Eurofins. After the digestion, DNA was run on a 0.8% agarose gel in 1X tris-acetate EDTA buffer (TAE) at 110 V for 1 h to separate NGF bands from the pEX-A2 backbone band. Then NGF containing gel fragments was cut by a scalpel and placed into a microcentrifuge tube. For successful ligations, NGF fragment was then purified from agarose gel using advanced QIAEX II® Gel Extraction Kit. After that, PPMN511A1 (V) and purified NGF fragment (I) were ligated using T4 DNA Ligase at 1:3 molar ratio of V: I at 22 °C for 3 h, followed by inactivation at 70 °C for 10 min. The ligation products were transformed into competent ZYCY10P3S2T *E. coli*, and eight colonies were selected for screening from the agar plates with kanamycin (50 µg mL^−1^). The NGF fragment was inserted into the PP (V) at a position downstream of the CMV promoter. The new generated plasmid is the PPMN511A1-NGF DNA (PP NGF, 7.7 kbp). The structure of the PP NGF was determined by restriction digestion and electrophoresis analysis on 1% agarose gels.

### MC NGF production

Highly pure MC NGF (3.7 kbp) under the control of CMV promoter was produced as following: On the first day, 20 µL of transformed ZYCY *E. coli* with constructed PP NGF were cultured in 5 mL of lysogeny broth (LB) media contains kanamycin (50 µg mL^−1^) at 37 °C under shaking (250 rpm) for 5 h (starting culture). Following which, 400 mL of terrific broth (TB) with kanamycin (50 µg mL^−1^) was inoculated with 100 µL of the TB from the starting culture media and incubated at 37 °C and 250 rpm shaking overnight. The next day, around 416 mL of MC Induction Mix (I.M.) (400 mL of LB, 16 mL of 1 N NaOH and 420 μL of 20% l-arabinose) was added and the culture media was incubated at 31 °C and 250 rpm shaking for 7 h. Next day, the bacterial cells contain MC NGF were centrifuged at 4149 x *g* and the plasmid was purified following standard procedures. The structure of the PP NGF and MC NGF were determined by restriction digestion and electrophoresis analysis on 1% agarose gels.

### Reporting summary

Further information on research design is available in the Nature Research Reporting Summary linked to this article.

## Supplementary information

Supplementary Information

Reporting Summary

## Data Availability

All data relevant to this study are available from the corresponding author upon reasonable request.
