# Supplementary material for: Highly branched poly(β-amino ester) delivery of minicircle DNA for transfection of neurodegenerative disease related cells
Source: Nat Commun. 2019 Jul 24;10:3307. doi: 10.1038/s41467-019-11190-0 (PMC6656726; doi:10.1038/s41467-019-11190-0)
Supplement: Supplementary file 1 — Supplementary Information [file 41467_2019_11190_MOESM1_ESM.pdf]

Supplementary Information

Highly branched poly(β-amino-ester) delivery of minicircle DNA for transfection of neurodegenerative related cells

Liu et al.

Table of Contents

SUPPLEMENTARY METHODS 3
SUPPLEMENTARY TABLE 1. 10
SUPPLEMENTARY TABLE 2. 11
SUPPLEMENTARY TABLE 3. 12
SUPPLEMENTARY TABLE 4. 13
SUPPLEMENTARY TABLE 5. 14
SUPPLEMENTARY FIGURE 1 15
SUPPLEMENTARY FIGURE 2 16
SUPPLEMENTARY FIGURE 3 17
SUPPLEMENTARY FIGURE 4 18
SUPPLEMENTARY FIGURE 5 20
SUPPLEMENTARY FIGURE 6 21
SUPPLEMENTARY FIGURE 7 22
SUPPLEMENTARY FIGURE 8 23
SUPPLEMENTARY FIGURE 9 24
SUPPLEMENTARY FIGURE 10 25
SUPPLEMENTARY FIGURE 11 26
SUPPLEMENTARY FIGURE 12 27
SUPPLEMENTARY FIGURE 13 28
SUPPLEMENTARY FIGURE 14 29
SUPPLEMENTARY FIGURE 15 30
SUPPLEMENTARY FIGURE 16 31
SUPPLEMENTARY FIGURE 17 32
SUPPLEMENTARY FIGURE 18 33
SUPPLEMENTARY FIGURE 19 34
SUPPLEMENTARY FIGURE 20 35
SUPPLEMENTARY FIGURE 21 36
SUPPLEMENTARY FIGURE 22 37
SUPPLEMENTARY FIGURE 23 38
SUPPLEMENTARY FIGURE 24 39
SUPPLEMENTARY FIGURE 25 40
SUPPLEMENTARY FIGURE 26 41
SUPPLEMENTARY FIGURE 27 42
SUPPLEMENTARY FIGURE 28 43
SUPPLEMENTARY FIGURE 29 44
SUPPLEMENTARY FIGURE 30 45
SUPPLEMENTARY FIGURE 31 46
SUPPLEMENTARY REFERENCES 47

Supplementary Methods

Materials

For polymer synthesis and characterization, 4-amino-1-butanol (S4, A2 type monomer), trimethylolpropane triacrylate (TMPTA, B3 type monomer), bisphenol A ethoxylate diacrylate (BE, C2 type monomer), 1,11-Diamino-3,6,9-trioxaundecane (DT, end-capping agent), 1H-pyrazole-1-carboxamidine hydrochloride (HPCH), N,N-diisopropylethylamine (DIPEA), deuterated chloroform ($CDCl_3$) and lithium bromide (LiBr) were purchased from Sigma-Aldrich. Solvents dimethyl sulfoxide (DMSO), dimethylformamide (DMF), acetone and diethyl ether were obtained from Fisher Scientific. Sodium acetate buffer solution (pH = 5.2 ± 0.1, 3 M) obtained from Aldrich was diluted to 0.025 M prior to use. Bovine serum albumin (BSA), agarose and tris-acetate EDTA (TAE) buffer were purchased from Sigma. Disufide monomer disulfanediylbis(ethane-2,1-diyl) diacrylate (DSDA) was prepared according to previous report[1]. For gene transfection, the cell culture media, fetal bovine serum (FBS), trypsin-EDTA and Hank's balanced salt solution (HBSS), phosphate buffered saline (PBS) and 4′,6-diamidino-2-phenylindole (DAPI) were purchased from Life Technologies. Alexa Fluor 594 was purchased from Thermo Fisher Scientific. QIAEX II® Gel Extraction Kits and TE buffer were obtained from Qiagen. T4 DNA Ligase was purchased from Promega. Commercial transfection reagents branched polyethyleneimine (PEI, M_w = 25 kDa) (Sigma), Lipofectamine 2000 (Lipo2k) and Lipofectamine 3000 (Lipo3k) (Life Technologies), SuperFect (Qiagen), FuGENE (Promega) and Xfect (Clontech) were used as per manufacturers' protocols. Green fluorescent protein plasmid (pCMV-GFP) was purchased from Aldevron. Cell secreted Gaussia princeps luciferase plasmid (pCMV-Gluc) were obtained from New England Biolabs. NGF plasmid pEX-A2-NGF (2.4 Kbp) was constructed by Eurofins. BioLux™ Gaussia luciferase assay kits, enzymes XbaI and BamHI obtained from New England Biolabs and alamarBlue® from Invitrogen were used as per protocols. Human beta-NGF protein were purchased from Sino Biological. DNA ladders was purchased from Thermo Fisher Scientific. Human beta-NGF ELISA Kits were purchased from Sigma and used as per the manufacturer's protocol.

Instrumentation

Chemical structure and composition of polymers were verified with ^1H NMR spectra, which was performed on a 400 MHz Varian NMR spectrometer. Molecular weight (M_w and M_n) and dispersity ($Đ$) of polymers were measured with gel permeation chromatograph (GPC) which was equipped with a triple-detector [a refractive index detector (RI), a viscometer detector (VS DP) and a dual angle light scattering detector (LS 15° and LS 90°)]. GPC measurements were conducted at 50 °C

with DMF (plus 0.1% LiBr) as elution at a flow rate of 1 mL/min. Polyplex size and zeta potential were measured on a Zetasizer (Nano Series Nano-2590, Malvern) with a scattering angle of 90 ^{o}C producing a wavelength of 633 nm.

LPAE and HPAE-3 degradation

LPAE and HPAE-3 were dissolved in DMSO at a concentration of 100 mg/mL and then diluted with PBS (pH = 7.4, 0.01 M) to 10 mg/mL. The solutions were kept stirring at 37°C. At the time points of 6, 12, 24 and 48 h, 1 mL of the solution was taken out and freeze-dried immediately and then dissolved in DMF and filtered with a 0.2 μm filter. Molecular weight was measured with GPC. The percentage of degradation was defined as the molecular weight of the degraded polymers divided by the molecular weight of the original polymer[2].

Polyplex preparation

HPAE/DNA polyplexes were prepared according to previous published procedures with slight modification[3-6]. HPAEs were first dissolved in DMSO (for molecular biology) by vortexing to give 100 mg/mL DMSO stock solutions and kept at -20 °C for the following studies. DNA was diluted with TE buffer to 1 mg/mL and kept at 4 °C for the following studies. The HPAE/DNA weight ratio (w/w) used in this work was 10:1 and 20:1. Taking the use of 1 μg DNA for polyplex preparation as an example: 1 μL DNA stock solution was added to 19 μL sodium acetate (pH 5.2, 0.025 M) and mixed by vortexing for 3-5 seconds. According to the w/w ratio of 10:1 or 20:1, 0.1 or 0.2 μL DMSO stock solution was added to 19.9 or 19.8 μL sodium acetate (pH 5.2, 0.025 M) and mixed by vortexing for 10-15 seconds to make sure the HPAE was dissolved completely in sodium acetate. Then, the HPAE solution was added into the DNA solution and mixed by vortexing for 10-15 seconds. The mixed solution was kept undisturbed for 10-15 minutes to allow HPAE/DNA polyplex formation. After that, dependent on the specific application, the polyplex solution was diluted with different media. When other amounts of DNA were used for polyplex preparation, the volume of the HPAE DMSO stock solution and sodium acetate buffer was adjusted accordingly.

DNA binding affinity of HPAEs

DNA binding affinity of HPAEs was quantified utilizing Picogreen assays. Briefly, polyplexes were prepared as above and 0.5 μg DNA was used for each sample. Then picogreen solution was added into the polyplex solution, the mixture was incubated for 5 minutes followed by the addition of 200 μL DMEM media. The mixture was then transferred to a black 96-well plate. Fluorescence of the solution was measured at a 490 nm excitation wavelength and a 535 nm emission wavelength.

The mixture of DMEM, DNA and Picogreen was used as control.

Transmission electronic microscopy (TEM) characterization of polyplex morphologies

To characterize polyplex morphologies, LPAE, HPAESS-3, HPAESS-4 or HPAESG-1 and DNA were diluted in sodium acetate and then mixed in equal volumes (1 µg DNA, w/w = 20:1). After 15 min incubation, the polyplexes were centrifuged at high speed for 1 min and then washed twice with deionized water. Afterwards, the polyplexes were re-suspended in water, 5 µL of polyplex solution was dropped on a holey carbon film on 200 mesh copper grids and freeze dried for 1 hour. Samples were observed with TEM (FEI Tecnai 120) with an accelerating voltage of 120 kV.

MC GFP preparation

The production of MC GFP was carried out according to our previous report[7]. Briefly, the parental plasmid purchased from Systems Biology MN511A1 was cultured in ZYCY10P3S2T E.coli (Systems Biology) and with the addition of L-Arabinose and a lowering of temperature (30°C), the minicircle with green fluorescent protein sequence was produced (MC GFP). The MC GFP was harvested from the bacterial culture with a Qiagen DNA prep kit.

Cell culture

The human-derived renal proximal tubular cell line HKC8 (ATCC), human cervical cancer cell line HeLa (ATCC), neuro-inhibitory astrocyte cell line Neu7 (kind gift from Prof James Fawcett, University of Cambridge), pheochromocytoma cells PC12 (ATCC) and rat adipose-derived stem cells rADSC (extracted from rat adipose tissue) were cultured in Dulbecco's modified Eagle Medium (DMEM) with 10% FBS and 1% Penicillin/Streptomycin (P/S). The human primary astrocytes (ScienCell Research Laboratory) were maintained in 50% DMEM/50% F12 Ham media with 10% FBS and 1% P/S. Human adipose derived mesenchymal stem cells hADSC (Invitrogen) were cultured in MesenPRO RS medium with basal medium, growth supplement and 1% P/S. All cells were cultured at 37°C, 5% CO_2 in a humid incubator under standard cell culture techniques. HeLa cells were chosen because they are frequently used in evaluating the gene transfection efficiency of non-viral gene vectors, the cells were authenticated by the vendor by morphology check and growth curve analysis, and tested for mycoplasma contamination regularly.

Cytotoxicity of the degraded byproducts of HPAE-3 and LPAE

Cytotoxicity of the degraded byproducts of HPAE-3 and LPAE were tested using HKC8 cells. Cells were seeded in 96-well plates at a density of 3×10^4 cells per well in 100 µL of cell culture media

and cultured until 80-90% confluence. The degraded byproducts of HPAE-3 and LPAE were dissolved in DMSO to 500 mg/mL. And then, according to the final concentration, the required amount of byproducts-containing DMSO solution was added into the cell culture mediate directly. After another 24 hours of incubation, the cell viability was measured using the alamarBlue reduction assay as per manufacturer's protocol. Briefly, cell culture media was removed, cells were washed with HBSS twice, and then 100 µL 10% alamarBlue HBSS solution was added and the cells were incubated for another 2 hours. Next, the alamarBlue solution was transferred to a black flat bottomed 96-well plate. Fluorescence was measured using the standard protocol. Cells without any treatment were set as 100% viable.

Evaluation of the gene transfection activity of HPAEs, HPAESSs and HPAESGs

One day prior to transfection, cells were seeded in 96-well plates at a density of 1~3 × 10^4 cells per well in 100 µL of cell culture media and cultured until 70-80% confluence. rADSC and hADSC used for transfection were under passage six, Neu 7 and primary astrocytes used for transfection were under passage five. 0.5 µg DNA per well was applied in HKC8 and HeLa, while 0.25 µg DNA per well was applied in rADSC, hADSC, Neu7 and primary astrocytes. To prepare polyplexes, HPAEs were dissolved in DMSO to 100 mg/mL as stock solutions, and then according to polymer to DNA weight ratios (w/w), varying stock solutions were diluted to 10 µL sodium acetate buffer. Gluciferase DNA was diluted to 0.05 mg/mL with sodium acetate buffer. Afterwards, the polymer solutions were added into the DNA solutions, vortexed, then kept standing for 10-15 minutes to allow polyplex formation. 100 µL cell culture media was then added and mixed by pipetting up and down several times. The cell culture media in the 96-well plates was aspirated with a multi-channel pipette, and the polyplex solution was added quickly. 4 hours later, the media in the wells were replaced with 100 µL fresh media; the cells were cultured for another 44 hours. The commercial transfection reagent, PEI was used at a w/w ratio of 4:1, whilst SuperFect, Lipofectamine 2000, FuGENE, Xfect and Lipofectamine 3000 were used according to the manufacturers' protocols. Analysis of gene transfection efficiency was performed utilizing BioLux™ Gaussia luciferase assay kit as per the provided protocols, with secreted Gluciferase activity directly detected in the cell supernatant and subsequently plotted in terms of relative light units (RLU). Cell viability after transfection was analyzed using the alamarBlue reduction assay. Briefly, after removing the supernatant, cells were washed with HBSS followed by addition of 100 µL 10% alamarBlue. 2 hours later, the alamarBlue solution was transferred to a black flat bottomed

96-well plate. Fluorescence was measured using the standard protocol. The cells treated only with DNA were plotted as 100% viable.

Cellular uptake of polyplexes

Gluciferase DNA was labeled with fluorescence dye Cy3 as per the standard protocols. HeLa cells were seeded in a 24-well plate at a density of 8,000 cells per well. Polyplex preparation and transfection were conducted as mentioned above with a DNA dosage of 0.5 μg per well. 4 hours post transfection, the cells were washed with HBSS buffer three times and collected for measurements. For visualization and imaging with fluorescent microscopy, cells were washed with HBSS buffer three times, fixed with paraformaldehyde for 20 minutes and then permeabilized with tritonx100 for another 15 minutes. DAPI was added to stain cell nucleus before visualizing with a fluorescent microscopy.

Polyplex endo/lysosomal escape study

Cells were seeded in 96 well plate at a density of 3×10^3 cells per well in 100 μL media. Using Cy3 labelled DNA, polyplxes were prepared and the gene transfection was carried out at w/w = 20:1. 4 hours later, the acidic endo/lysosomal vesicles of cells were stained with Lyso-Tracker Green at 37 ^{o}C for 1 minute. Cells were then imaged immediately using a fluorescent microscopy. Pearson Correlation Coefficient (PCC) of the cells after transfection with HPAE-3/DNA and LPAE/DNA polyplexes were calculated using ImageJ from three images.

Gene transfection HPAESG/MC DNA in ADSCs and astrocytes

To evaluate the effectiveness of the optimized HPAESG in combination with MCDNA in gene transfection, cells were seeded in 24-well plates at a density of 5×10^4 cells/well in 500 μL media and cultured until 70-80% confluence. Transfection was performed as mentioned above with 1 μg MC DNA per well. To demonstrate the superiority of MC DNA, a normally sized DNA (5.6 kbp) was also used to carry out all the transfection in the same manner for comparison. 48 hours post transfection, the GFP positive cells were visualized and imaged with a fluorescent microscope. The transfection efficiency was analyzed via flow cytometry. Cells were collected as per standard protocols, at least 8,000 cells were counted for each sample. Cells that received only DNA were used as controls.

HPAESG-1/MC NGF transfect astrocytes

The effectiveness of HPAESG-1 in combination with MC NGF to mediate the expression and

production of NGF in Neu7 and primary astrocytes was studied. The gene transfection parameters including cell density (5×10^4 or 2×10^5cells/well, 24 well plates), DNA dosage (1 or 2 μg/well), with or without serum and transfection time (24, 48 or 72 hours) were optimized systematically. Briefly, cells were seeded in 24-well plates and cultured until 80-90% confluence. Transfection in the presence of serum was performed as mentioned above. For serum free transfection, cells were first incubated with HPAESG-1/MC NGF polyplexes in the absence of serum for 4 hours, and then the cell culture media was replaced with 10% FBS containing media. PP NGF where the MC NGF was derived from was used as a control for comparison, and all the transfection was carried out in exactly the same manner. There is a GFP tag with the MC NGF and PP NGF, therefore, after 24, 48 and 72 hours, the GFP expression was observed with a fluorescence microscope and the transfection efficiency was quantified with flow cytometry. Cell viability was analyzed with alamarBlue assays. Meanwhile, the cell supernatants were collected and the NGF concentration was measured with enzyme-linked immunosorbent assay (ELISA) as per the manufacturer's protocol.

Bioactivity of NGF produced by astrocytes after transfection.

PC12 cells were seeded in 96-well plates at a density of 1×10^3 cells/well in 100 μL media and incubated overnight to attach. And then, the media on the cells was replaced with standard PC12 cell culture media (DMEM with a high glucose concentration, PC12 media group), standard Neu7 cell culture media (DMEM with a low glucose concentration, Neu7 media group), conditioned Neu7 cell culture media (media saved from Neu7 cells after transfection with HPAESG-1/MC NFG, the concentration of NGF was determined to be 26.9 ng/mL, Conditioned Neu7 media group) and standard PC12 cell culture media but with 50 ng/mL recombinant NGF (PC12+NGF group), respectively. Over the following two weeks, the media on the cells were changed with the corresponding media as mentioned above every 3 days. The outgrowth of neurites was checked with a fluorescent microscope daily. At day 7 and 14, the cells were washed with PBS slightly three times and then fixed with paraformaldehyde (PFA) following the standard fixation procedures. The cell nucleus and F-actin were stained with DAPI (blue) and Alexa Fluor 594 (red), as per the manufacturers' protocols, respectively. And then, the cells were observed with a fluorescent microscope to check the neurite outgrowth.

Direct transfection of PC12 with HPAESG-1/MC NGF

PC12 cells were seeded in 24-well plates at a density of 5×10^3 cells/well in 500 μL media. The next day, with 1 μg DNA per well, the cells were transfected with HPAESG-1/MC NGF or HPAESG-

1/PP NGF in the absence of serum as mentioned above. The cell culture media was changed every 3 days. One week post transfection, GFP expression and neurite outgrowth were checked with a fluorescent microscope, the efficiency of GFP and NGF expression was measured with flow cytometry and ELISA, respectively.

Statistical analysis

All transfection data were analyzed using GraphPad Prism version 8 (GraphPad Software). A one-way ANOVA was performed, followed by Dunnett method as the post hoc test. P values < 0.05 were considered to be statistically significant. Additionally, data of the GFP positive cell percentage was analyzed using paired Student's t - test. All transfection experiments were performed in quadruplicate unless otherwise stated, with error bars indicating ±SD.

Supplementary Table 1.

Monomer feed ratios for the synthesis of LPAE and HPAE.

	[TMPTA]:[BE]	TMPTA[a] g (mmol)	BE[a] g (mmol)	S4[a] g (mmol)	DT g (mmol)	DMSO[b] (mL)
LPAE	0:1	0	0.56 (1.20)	0.09 (1.0)	0.23 (1.2)	9
HPAE-1	1:13.5	0.02 (0.08)	0.51 (1.08)	0.09 (1.0)	0.23 (1.2)	9
HPAE-2	1:6.0	0.05 (0.16)	0.45 (0.96)	0.09 (1.0)	0.23 (1.2)	9
HPAE-3	1:3.5	0.11 (0.36)	0.59 (1.26)	0.13 (1.5)	0.35 (1.8)	10
HPAE-4	1:1.5	0.18 (0.60)	0.42 (0.90)	0.13 (1.5)	0.35 (1.8)	10
HPAE-5	1:0.5	0.36 (1.20)	0.28 (0.60)	0.18 (2.0)	0.46 (2.4)	12

a. For the synthesis of HPAEs, the total monomer concentration was 500 mg/mL in DMSO.

b. Volume of DMSO used to dilute the base polymers prior to end-capping with DT.

Supplementary Table 2.

Monomer feed ratio, polymer composition and structural information of LPAE and HPAE.

	Feed ratio	Composition				
	[TMPTA]:[BE][a]	[TMPTA]:[BE][b]	M_w (Da)[c]	M_n (Da)[c]	$Đ$[c]	α[d]
LPAE	0:1	0:1	12,043	4,764	2.53	0.63
HPAE-1	1:13.5	1:12.1	13,803	4,702	2.94	0.48
HPAE-2	1:6.0	1:5.2	15,288	5,201	2.94	0.46
HPAE-3	1:3.5	1:3.6	14,433	4,261	3.39	0.44
HPAE-4	1:1.5	1:1.58	20,874	3,070	6.80	0.37
HPAE-5	1:0.5	1:0.71	19,526	3,491	5.59	0.32

a. Calculated from feed ratios

b. Calculated from ^{1}H NMR spectra

c. Measured by RI detector

d. α: Mark-Houwink exponent, measured by VS DP detector

Supplementary Table 3.

Molecular weight and distribution of HPAE-3 base polymers from different batches

Batch	M_n (Da)	M_w (Da)	$Đ$
1	4027	8835	2.2
2	3895	8937	2.3
3	4036	9171	2.3
4	4044	8657	2.1
5	3973	8471	2.1
6	3892	8487	2.2
7	3736	8703	2.3

Supplementary Table 4.

Monomer feed ratio for the synthesis of HPAESS.

	TMPTA[a] g (mmol)	BE[a] g (mmol)	DSDA[a] (g, mmol)	S4[a] g (mmol)	DT g (mmol)	DMSO[b] (mL)
HPAESS-1	0.11(0.36)	0.57(1.22)	0.01(0.05)	0.13(1.5)	0.35(1.8)	10
HPAESS-2	0.11(0.36)	0.55(1.17)	0.02(0.09)	0.13(1.5)	0.35(1.8)	10
HPAESS-3	0.11(0.36)	0.51(1.08)	0.05(0.18)	0.13(1.5)	0.35(1.8)	10
HPAESS-4	0.11(0.36)	0.46(0.99)	0.07 (0.27)	0.13(1.5)	0.35(1.8)	9.5
HPAESS-5	0.11(0.36)	0.42(0.90)	0.09(0.36)	0.13(1.5)	0.35(1.8)	9.5

a. For the synthesis of HPAESSs, the monomer concentration is 500 mg/mL in DMSO.

b. Volume of DMSO used to dilute the base polymers prior to end-capping with DT.

Supplementary Table 5.

Monomer feed ratio, polymer composition and structural information of HPAESS.

	Feed ratio	Composition				
	[TMPTA]:[BE]:[DSDA][a]	[TMPTA]:[BE]:[DSDA][b]	M_w (Da)[c]	M_n (Da)[c]	$Đ$[c]	α[d]
HPAESS-1	1:3.4:0.13	1:3.9:0.18	13,158	3,302	3.98	0.48
HPAESS-2	1:3.3:0.25	1:3.8:0.25	14,124	3,162	4.47	0.48
HPAESS-3	1:3.0:0.50	1:3.8:0.34	15,908	3,047	5.22	0.48
HPAESS-4	1:2.8:0.75	1:3.3:0.38	16,052	3,423	4.69	0.46
HPAESS-5	1:2.5:1.70	1:3.3:0.56	14,077	2,712	5.19	0.47

a. Calculated from feed ratio

b. Calculated from ^{1}H NMR spectra

c. Measured by RI detector

d. α: Mark-Houwink exponent, measured by VS DP detector

Supplementary Figure 1

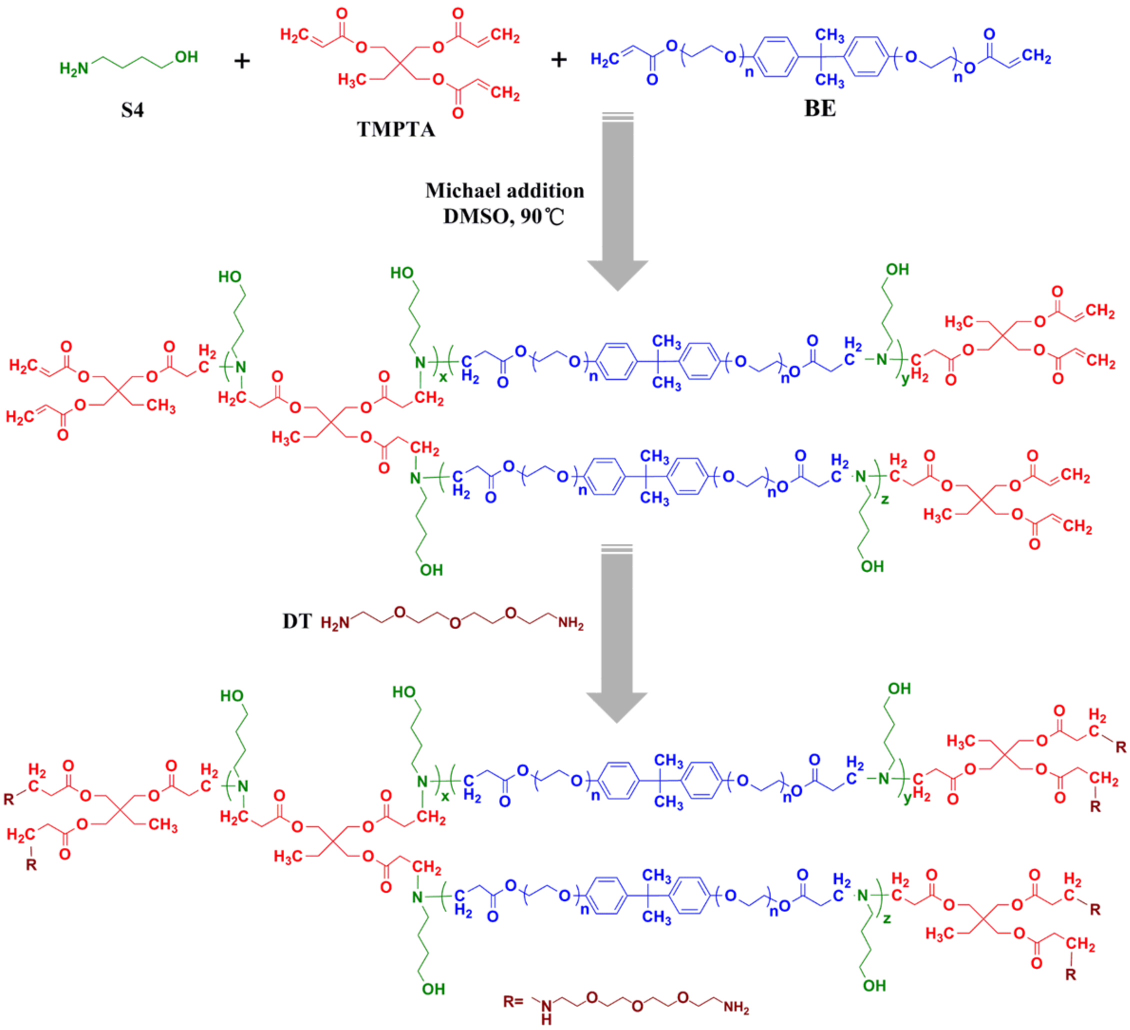

Synthesis of HPAEs via an "A2+B3+C2" Michael addition chemistry. S4, TMPTA and BE were copolymerized in DMSO at 500 mg mL^{-1} concentration to generate acrylate terminated based polymer, and then DT was used as end-capping agent to end-cap the base polymer to generate the HPAEs.

Supplementary Figure 2

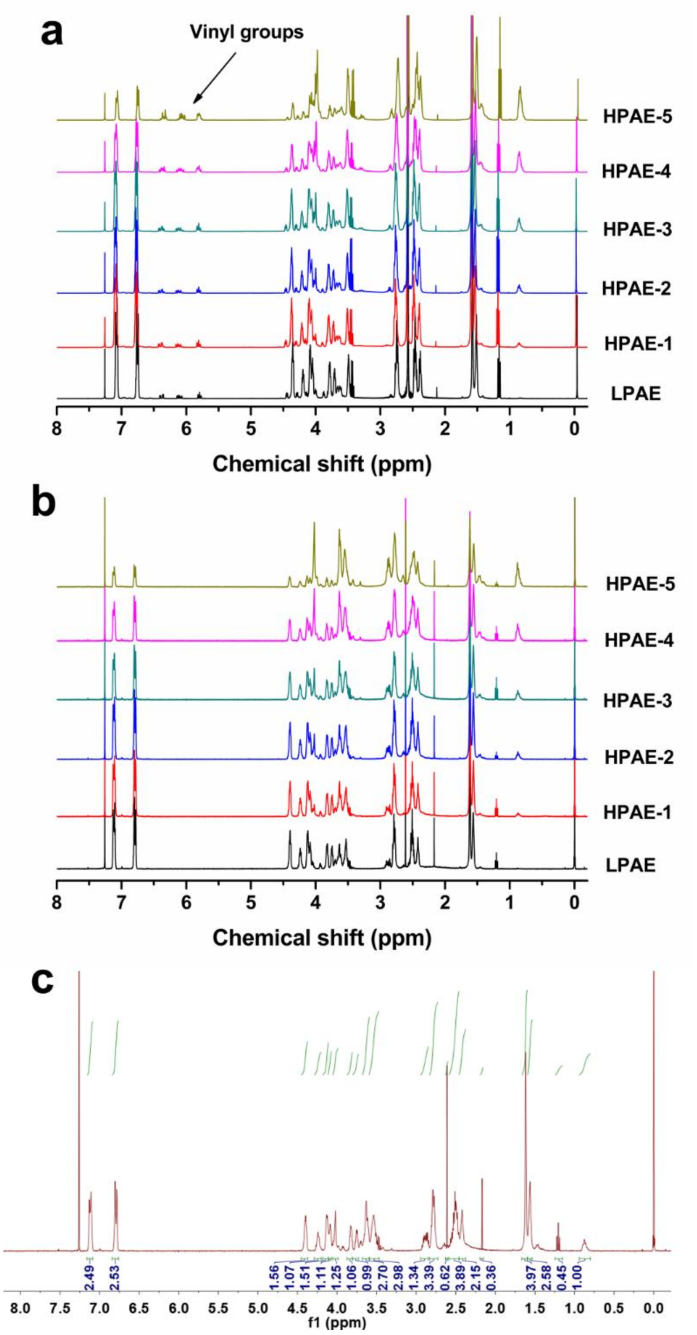

^{1}H NMR spectra of LPAE and HPAEs before and after end-capping. (a) ^{1}H NMR spectra of acrylate terminated HPAEs and LPAE base polymers. Spectra illustrated that prior to end-capping, at roughly 6.0 ppm, multiple signal peaks represent the vinyl groups in the base polymers. (b) ^{1}H NMR spectra of HPAEs and LPAE, it is clearly shown that after end-capping, the vinyl groups have disappeared. (c) ^{1}H NMR spectrum of HPAE-3 in $CDCl_3$ after end-capping.

Supplementary Figure 3

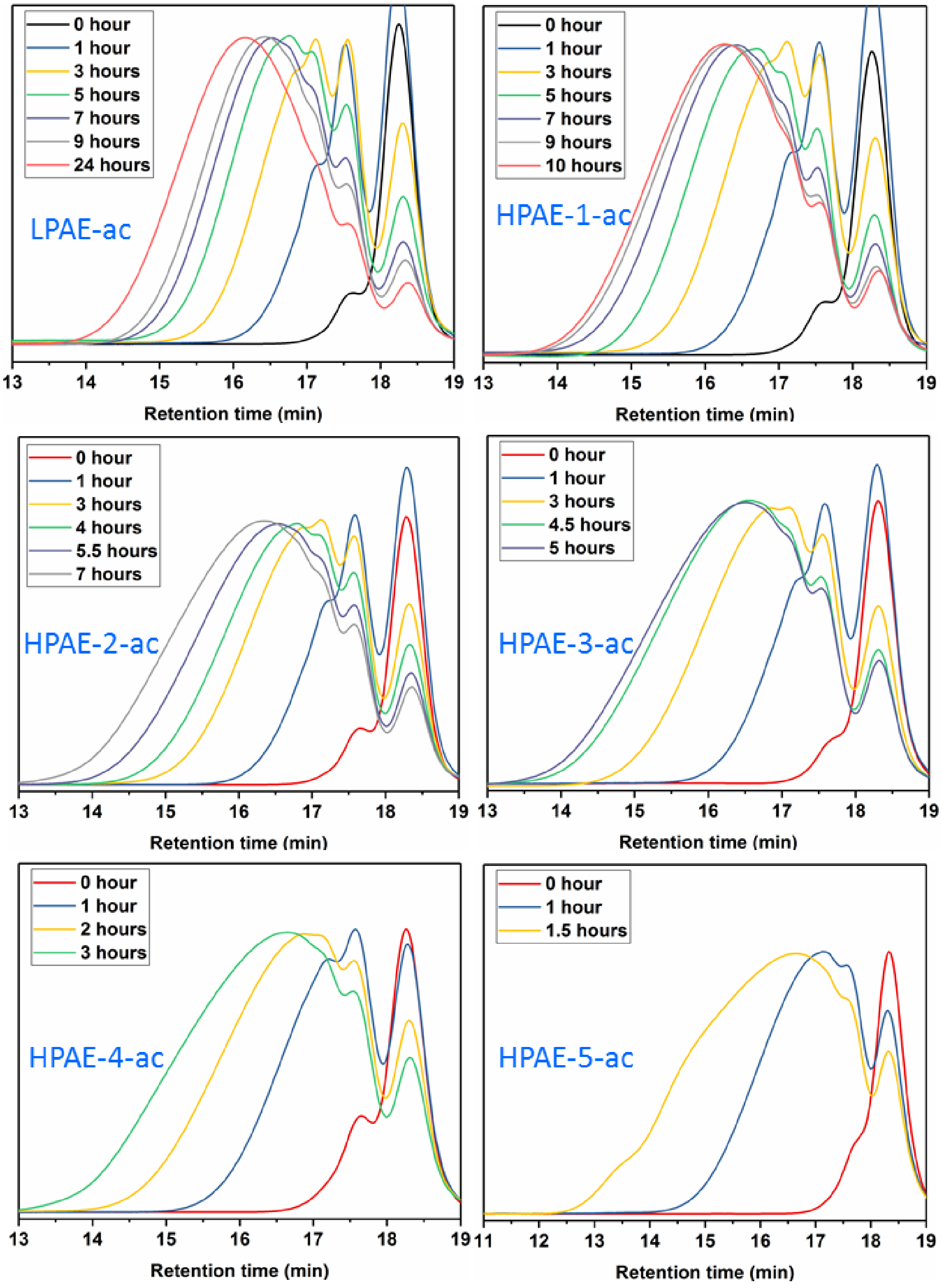

The evolution of GPC curves of LPAE and HPAE base polymers. Mass spectrometry is usually utilized to measure the molecular weight of small molecules. Since polymers show molecular weights of broad distribution, herein, GPC was used to characterize their molecular weights and dispersity.

Supplementary Figure 4

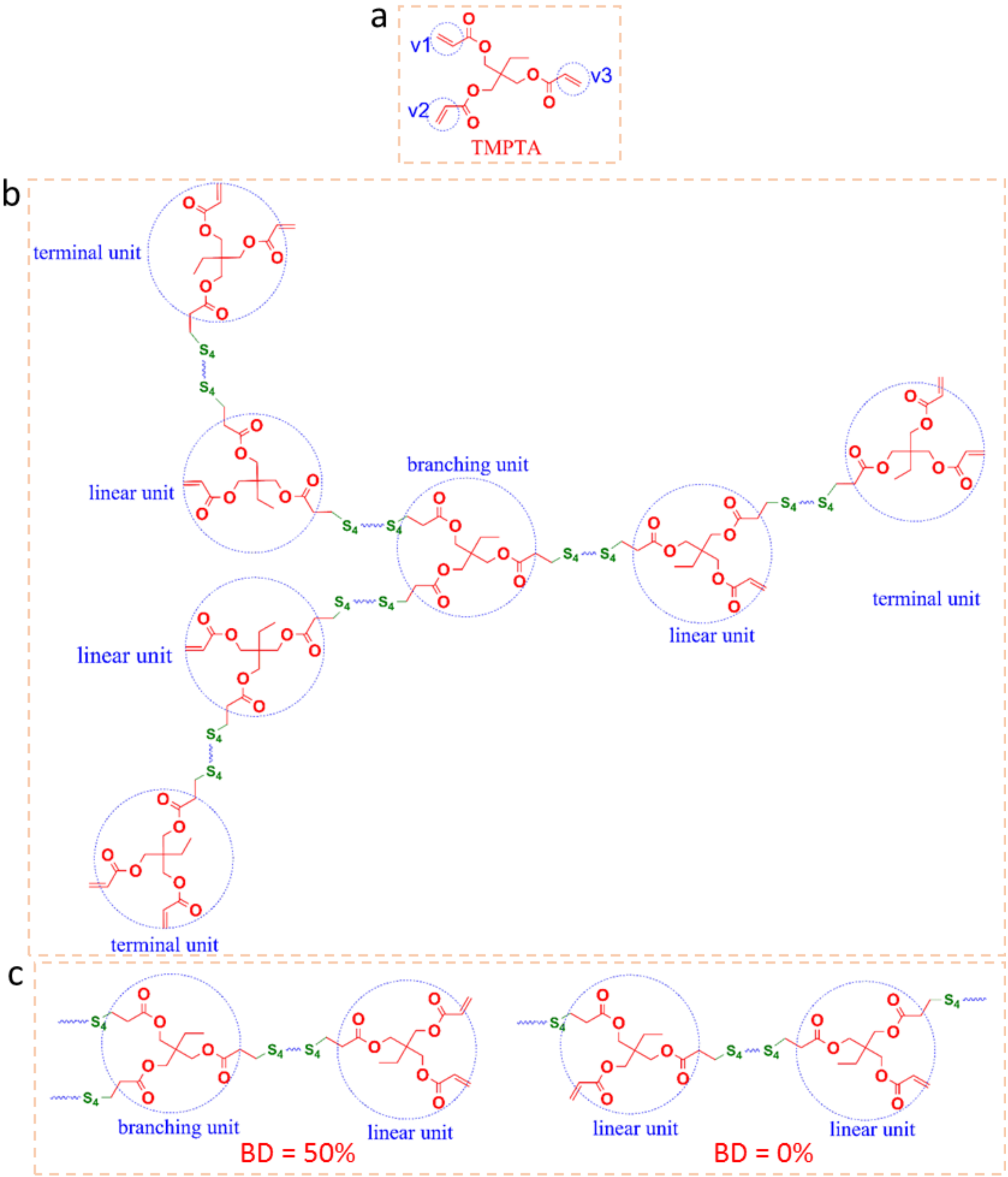

(a) Chemical structure of TMPTA, which contains three vinyl groups of the same chemical environment; (b) Schematic illustration of branching unit, linear unit and terminal unit in HPAE; (c) Illustration of HPAEs with the same vinyl group conversion but different BD.

In this work, TMPTA is used as the branching monomer, the branching degree (BD) can be defined as:

$$BD = \frac{number\ of\ TMPTA\ units\ with\ all\ the\ three\ vinyl\ groups\ consumed}{number\ of\ TMPTA\ units\ with\ one, two\ or\ three\ vinyl\ groups\ consumed} \times 100\%$$

As shown above (**Supplementary Figure 4a**), chemical environment of the three vinyl groups (v1, v2 and v3) in TMPTA are is exactly the same. Different vinyl group quantity (n = 1, 2 or 3) in the same TMPTA consumed by the amine S4 during the based polymer polymerization process would lead to formation of terminal unit (n = 1), linear unit (n = 2) or branching unit (n = 3) (**Supplementary Figure 4b**). Furthermore, even with the same overall vinyl group conversion, different numbers of branching units, linear units and terminal units could be formed (**Supplementary Figure 4c**), and thus it is not possible to calculate the BD (the NMR spectra of all the HPAE based polymers are shown in **Supplementary Figure 2a**).

Supplementary Figure 5

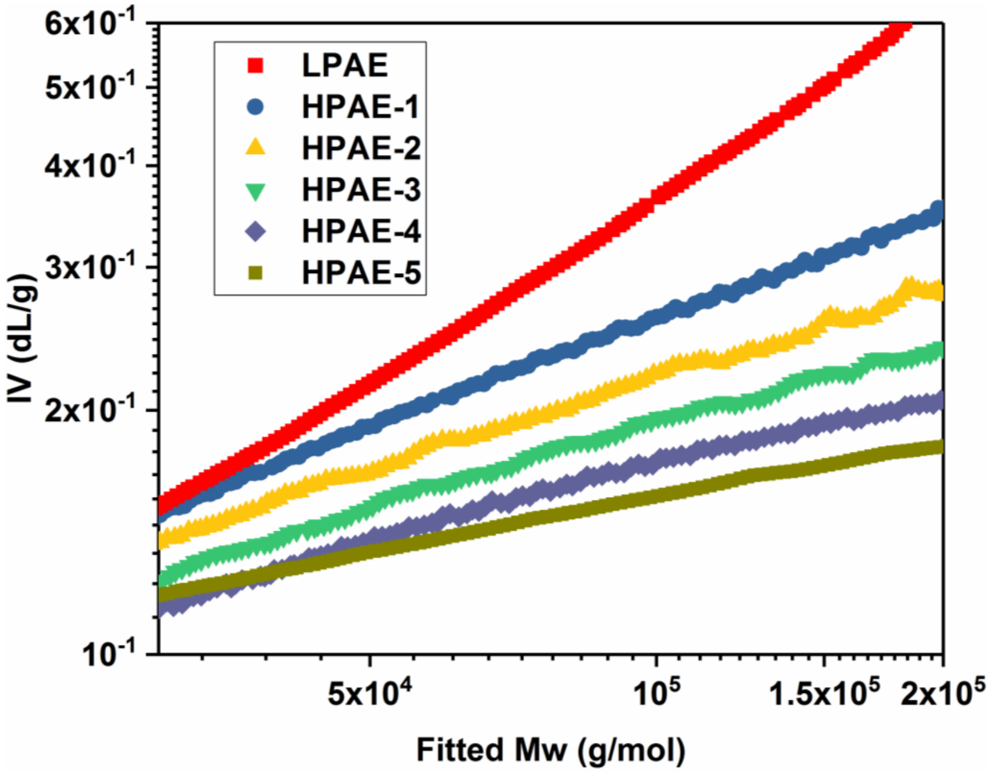

Mark-Houwink plots of LPAE and HPAEs.

Supplementary Figure 6

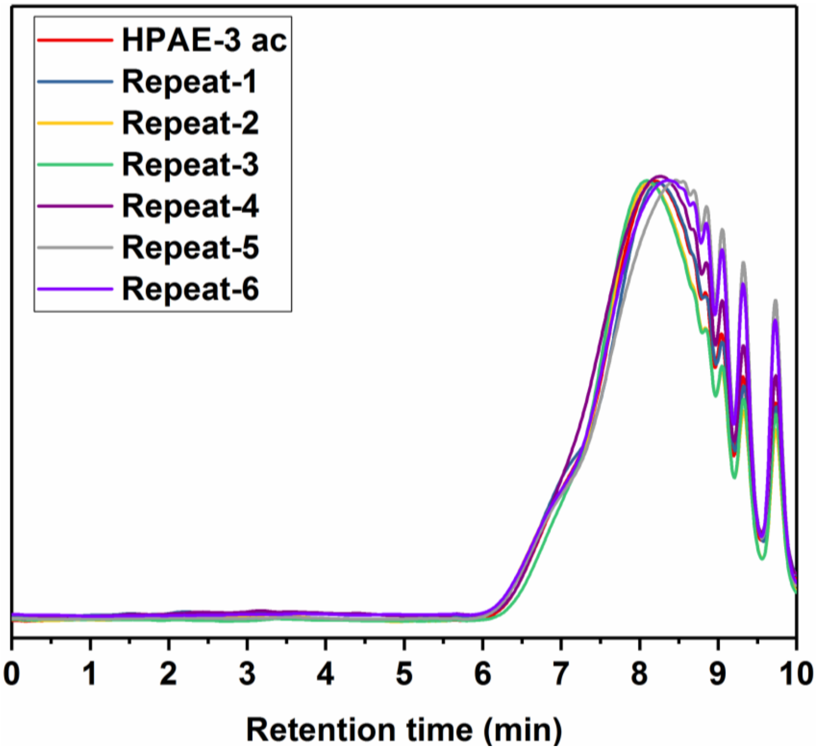

GPC traces of HPAE-3 base polymers synthesized from seven different batches.

Supplementary Figure 7

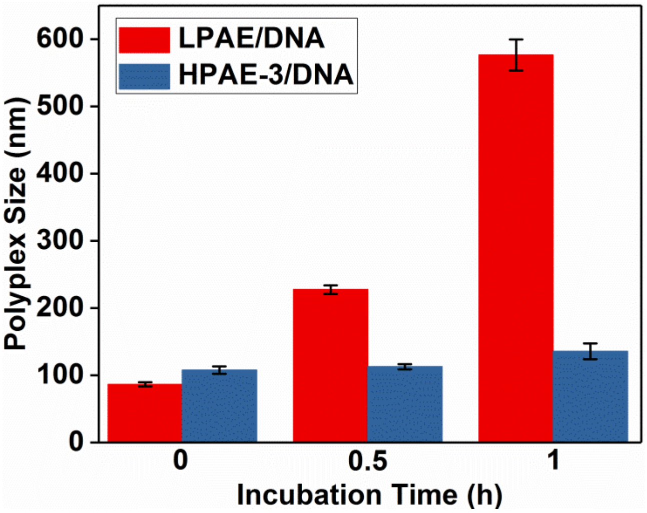

Sizes of LPAE/DNA and HPAE-3/DNA polyplexes (w/w = 20:1) after incubation with 0.5% BSA solution at different time points. Data are shown as average ± SD; n = 4.

Supplementary Figure 8

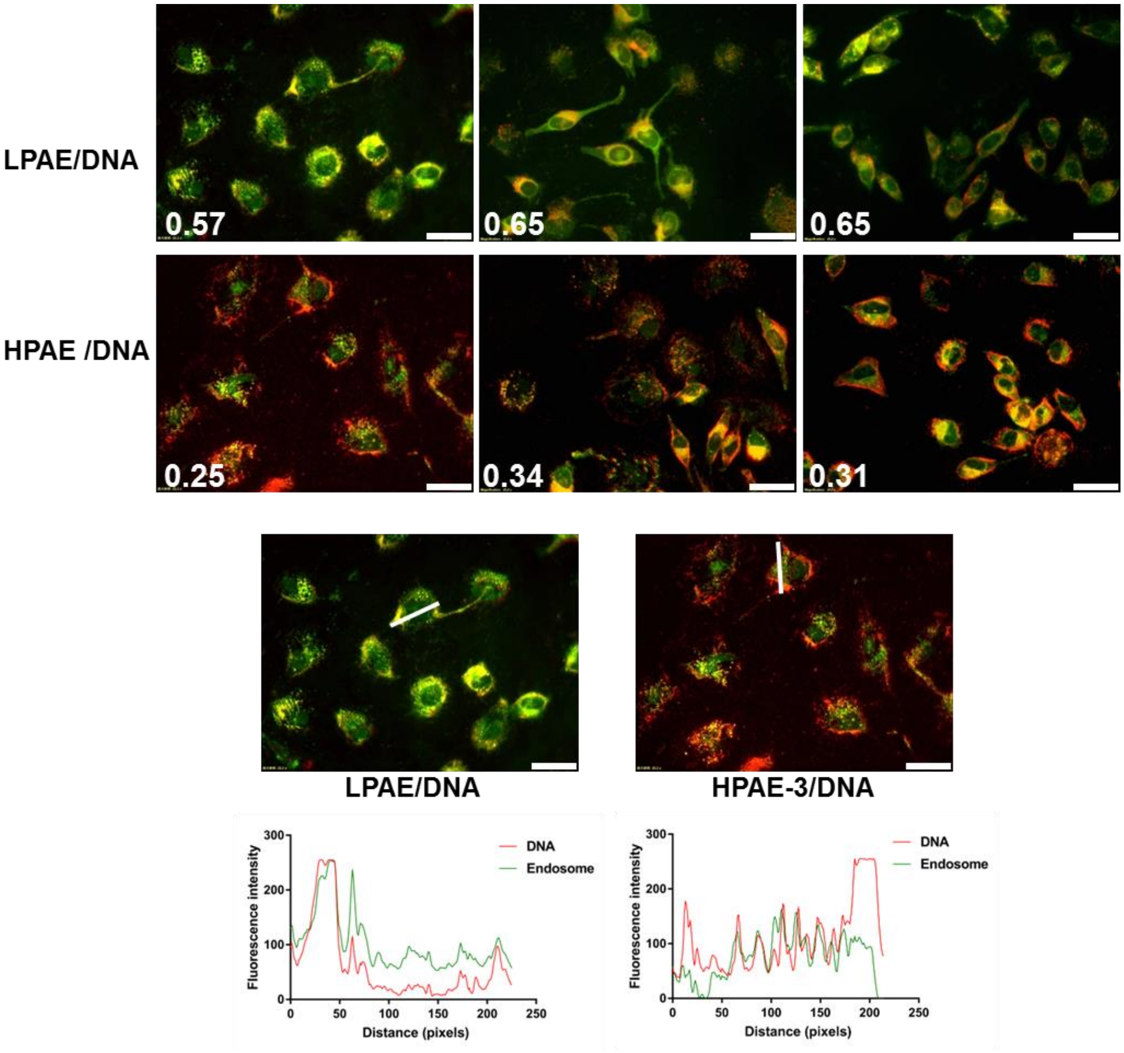

Comparison of the endo/lysosomal escape ability of the HPAE-3/DNA polyplexes and LAPE/DNA polyplexes by quantifying the PCC with ImageJ, the PCCs were shown in the images in white, the scale bars represent 50 μm. Plot profiles of the representative cells (marked in white line) are also shown.

Supplementary Figure 9

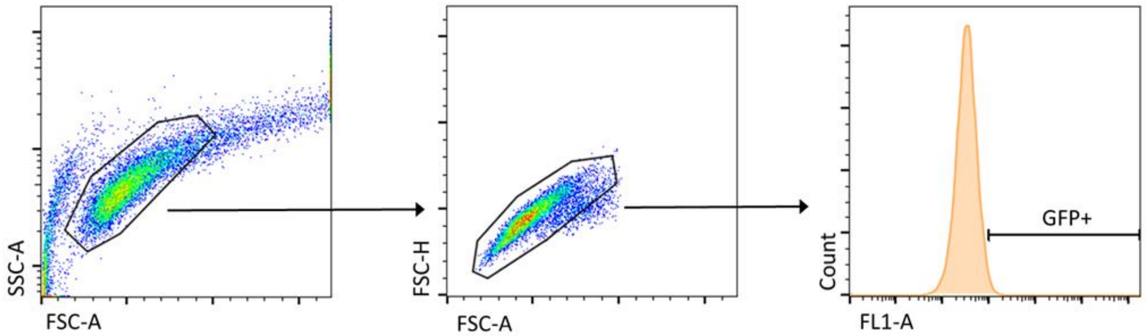

Gating strategy used for sorting GFP positive cells after gene transfection presented on **Figure 3c, 3d, 5d, 5e, 7d, 7e, 8a, 9c, Supplementary Figure 10**, and **Supplementary Figure 30b**.

Supplementary Figure 10

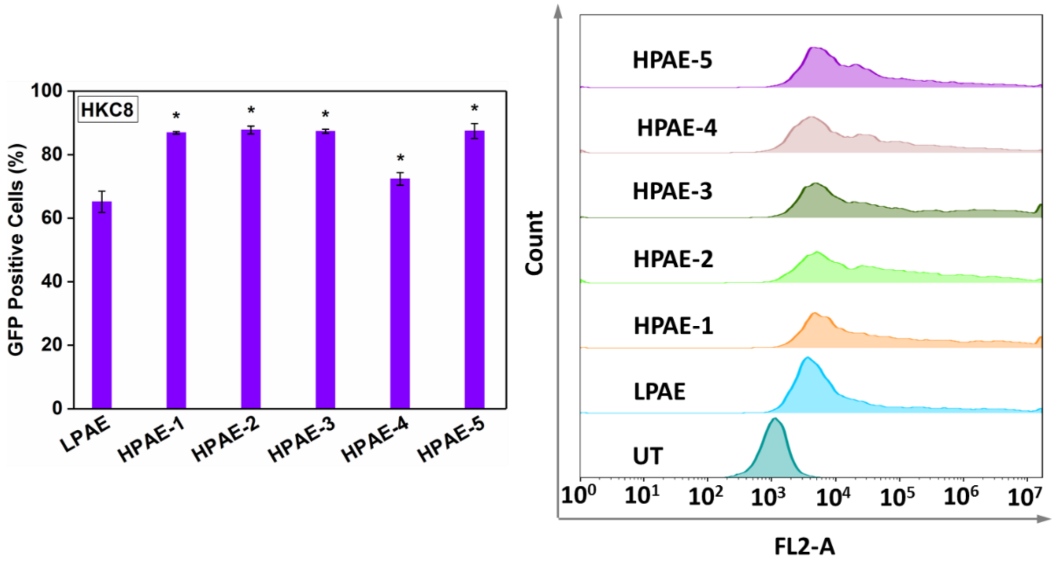

GFP expression of HKC8 cells after transfection quantified with the HPAE/DNA polyplex series quantified with flow cytometry. (a) The percentage of GFP positive cells after transfection, * indicates significantly higher transfection efficiency in comparison with the LPAE group; Data are shown as average ± SD; n = 4. (b) Representative histogram distributions of untreated cells (UT) and the ones after transfection with HPAEs at the w/w = 20:1.

Supplementary Figure 11

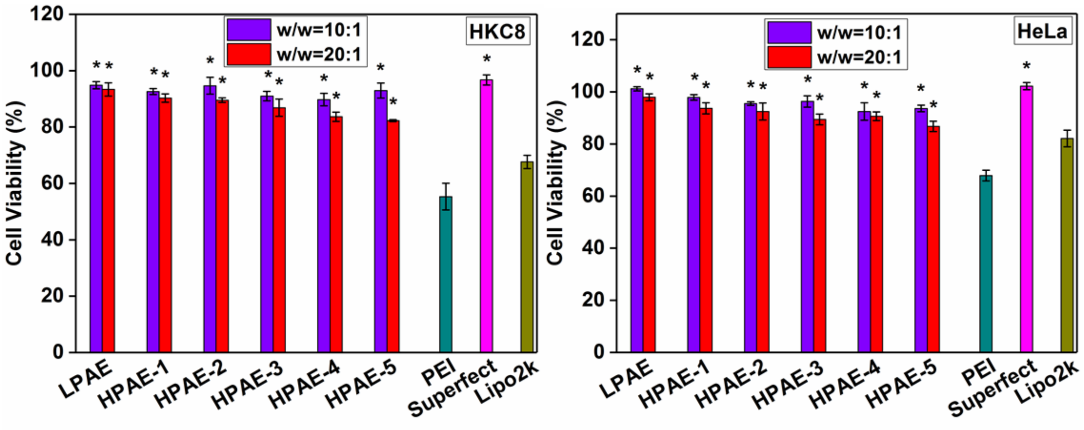

Cell viability after transfection with LPAE, HPAEs and commercial gene transfection reagents. Data are shown as average ± SD; n = 4.

Supplementary Figure 12

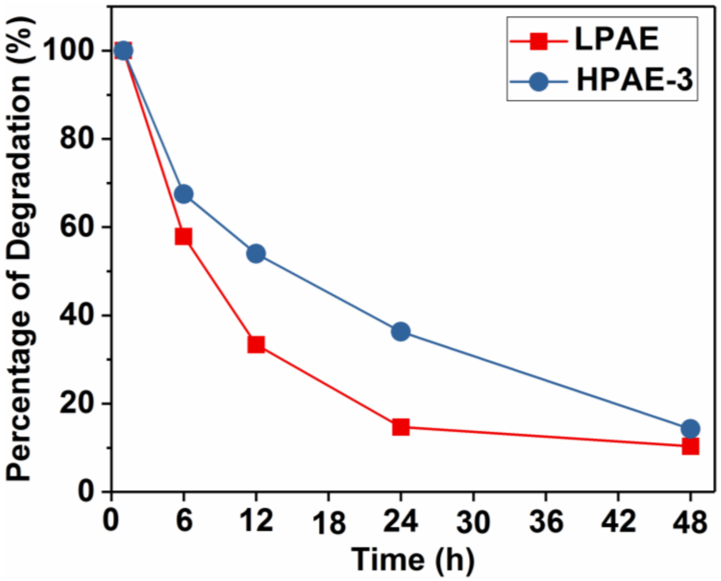

Degradation profiles of LPAE and HPAE-3 in PBS.

Supplementary Figure 13

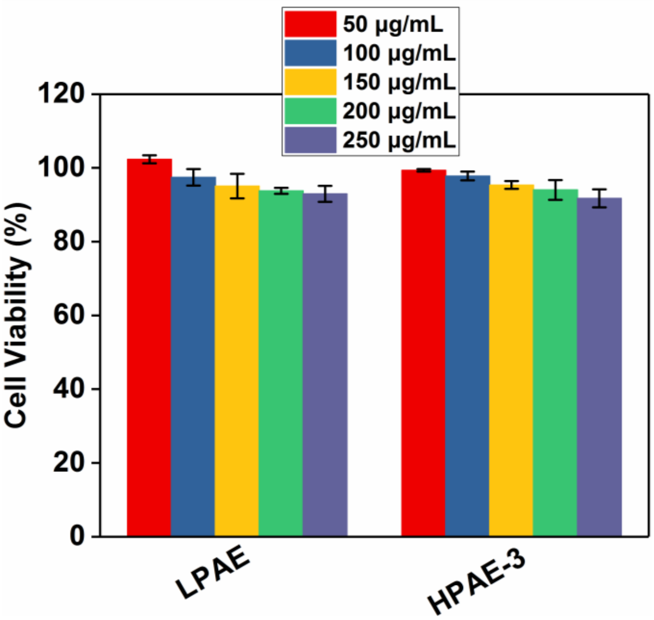

Viability of HKC8 cells after treatment with the degraded byproducts of HPAE-3 and LPAE at different concentrations. Data are shown as average ± SD; n = 4.

Supplementary Figure 14

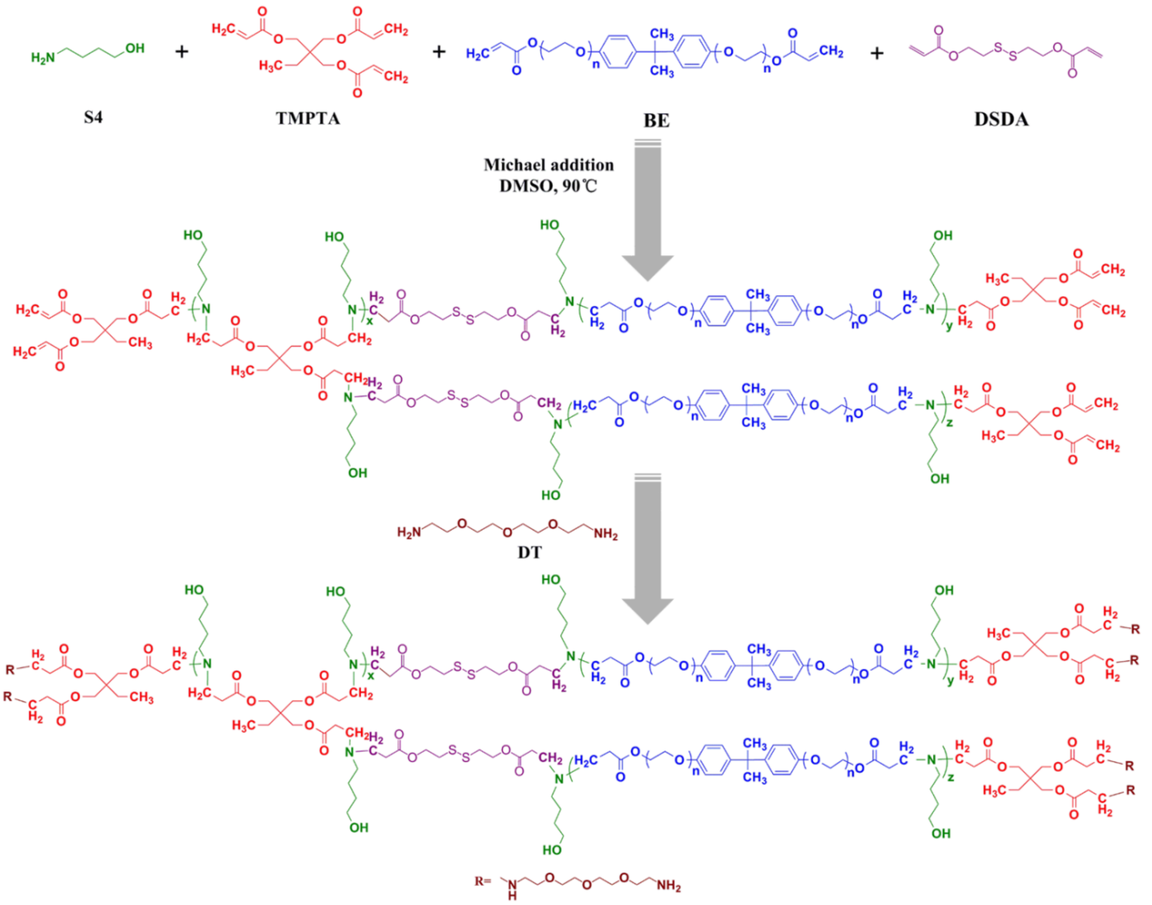

Synthesis of HPAESS via the "A2+B3+C2" Michael addition approach. S4, TMPTA, BE and DSDA were copolymerized first to generate acrylate terminated based polymer, and then DT was used to end-cap the base polymer to generate the HPAESS.

Supplementary Figure 15

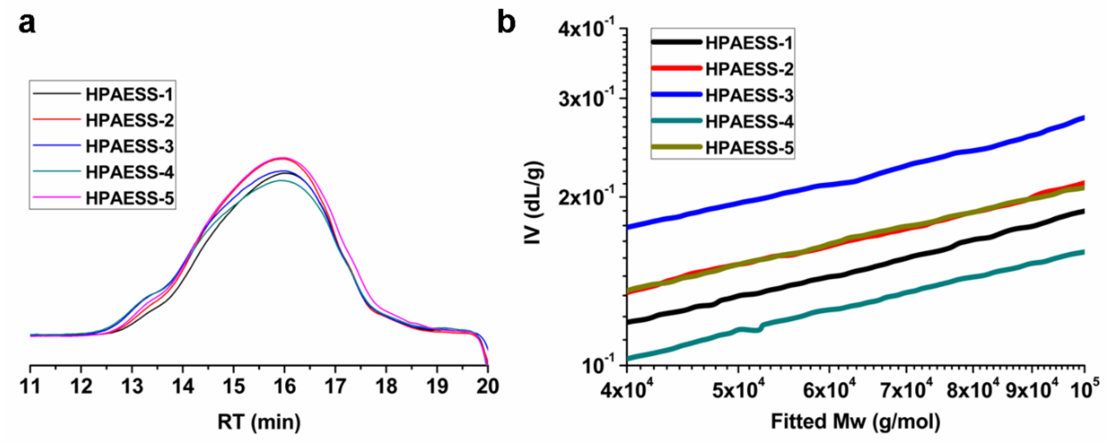

GPC traces (a) and Mark-Houwink plots (b) of HPAESS. All the five HPEASS have similar GPC traces which indicates their close molecular weight. The α values of all the HPAESS are below 0.5, indicating their highly branched structures.

Supplementary Figure 16

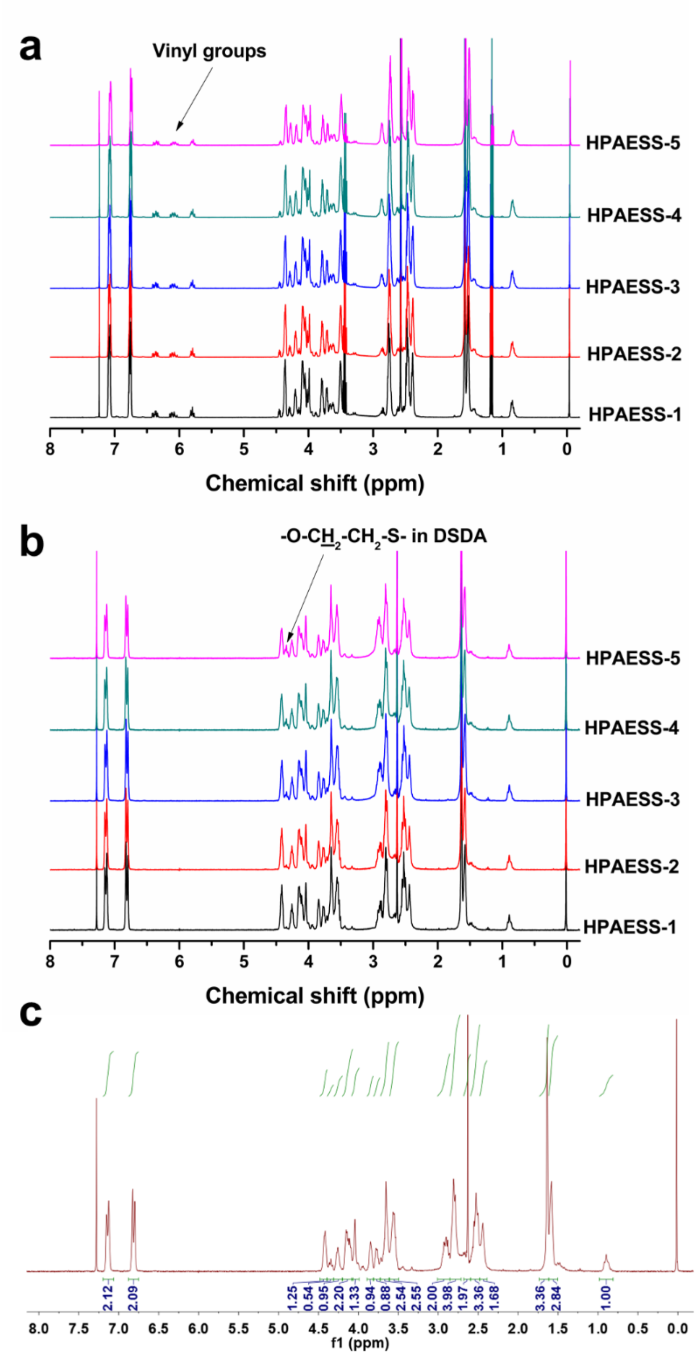

^{1}H NMR spectra of HPAESS before and after end-capping. (a) ^{1}H NMR spectra of HPAESS prior to end-capping. Prior to end-capping, at roughly 6.0 ppm, multiple signal peaks represented the vinyl groups left in the base polymers. (b) ^{1}H NMR spectra of HPAESS after end-capping, and the vinyl groups disappeared. The peak at 4.35 ppm was assigned to methylene of DSDA. (c) ^{1}H NMR spectrum of HPAESS-4 in $CDCl_3$ after end-capping.

Supplementary Figure 17

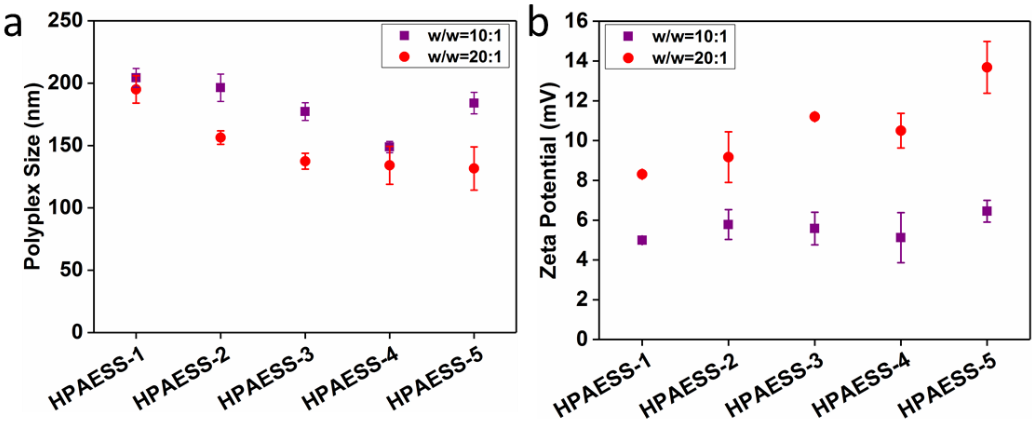

Size (a) and zeta potential (b) of the different HPAESS/DNA polyplexes at the w/w ratio of 10:1 and 20:1. Data are shown as average ± SD; n = 4.

Supplementary Figure 18

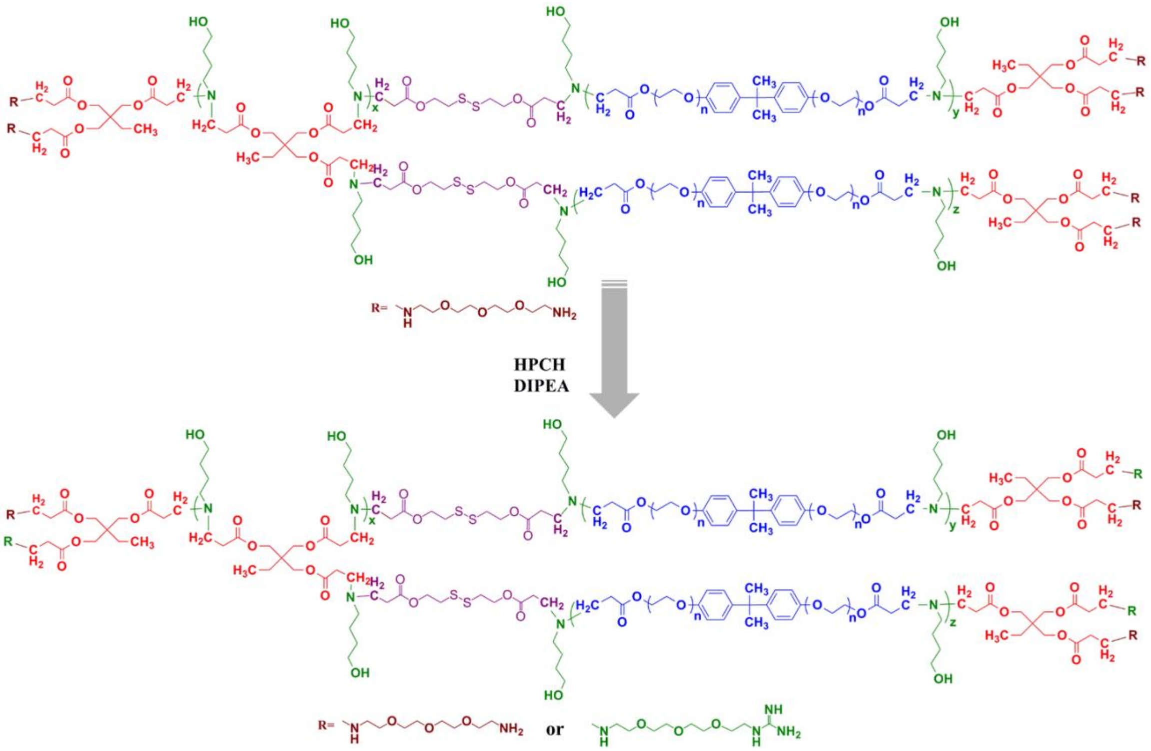

Guanidinylation of HPAESS to introduce multiple guanidine moieties at the peripheries to impart HPAESS enhanced cellular penetrating ability.

Supplementary Figure 19

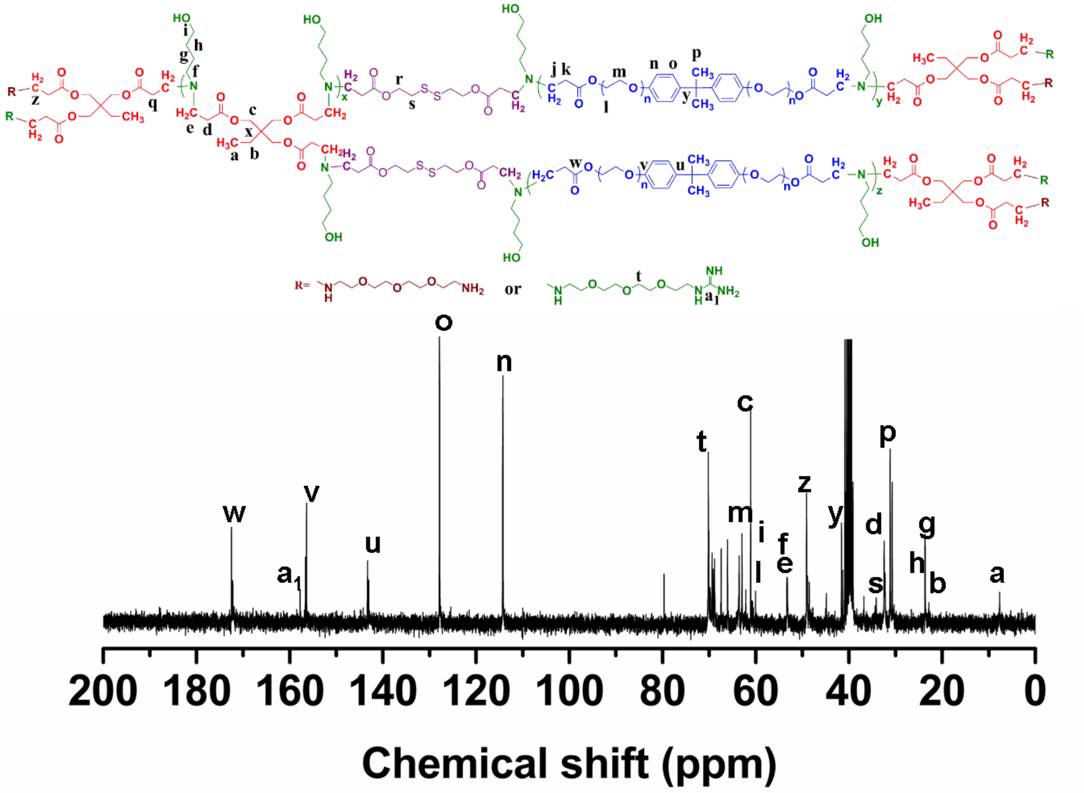

^{13}C NMR spectrum confirm the successful introduction of guanidine moieties to HPAESS. The peak at 157.8 ppm represented the carbon of guanidine moieties. ^{13}C NMR (DMSO-d6, ppm) δ 7.7, 22.9, 23.7, 26.6, 30.8, 31.2, 32.4, 34.2, 36.8, 39.2, 39.4, 39.7, 40.0, 40.3, 40.5, 40.8, 40.9, 41.3, 41.6, 44.9, 48.5, 48.9, 49.1, 53.2, 60.1, 61.1, 62.1, 63.0, 63.6, 66.1, 67.4, 68.8, 69.1, 69.4, 69.8, 70.1, 70.2, 79.7, 114.3, 127.9, 143.1, 143.3, 156.4, 156.6, 157.8, 172.2, 172.5.

Supplementary Figure 20

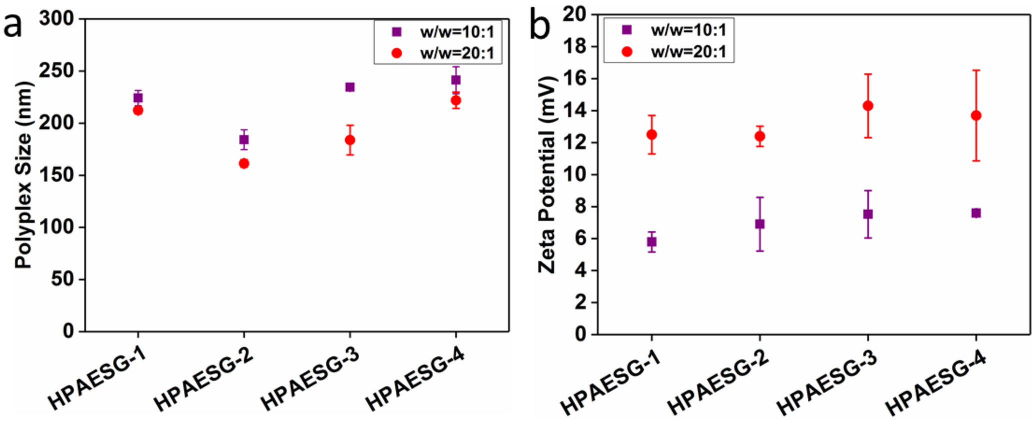

Size and zeta potential of the different HPAESG/DNA polyplexes at the w/w ratio of 10:1 and 20:1.

Data are shown as average ± SD; n = 4.

Supplementary Figure 21

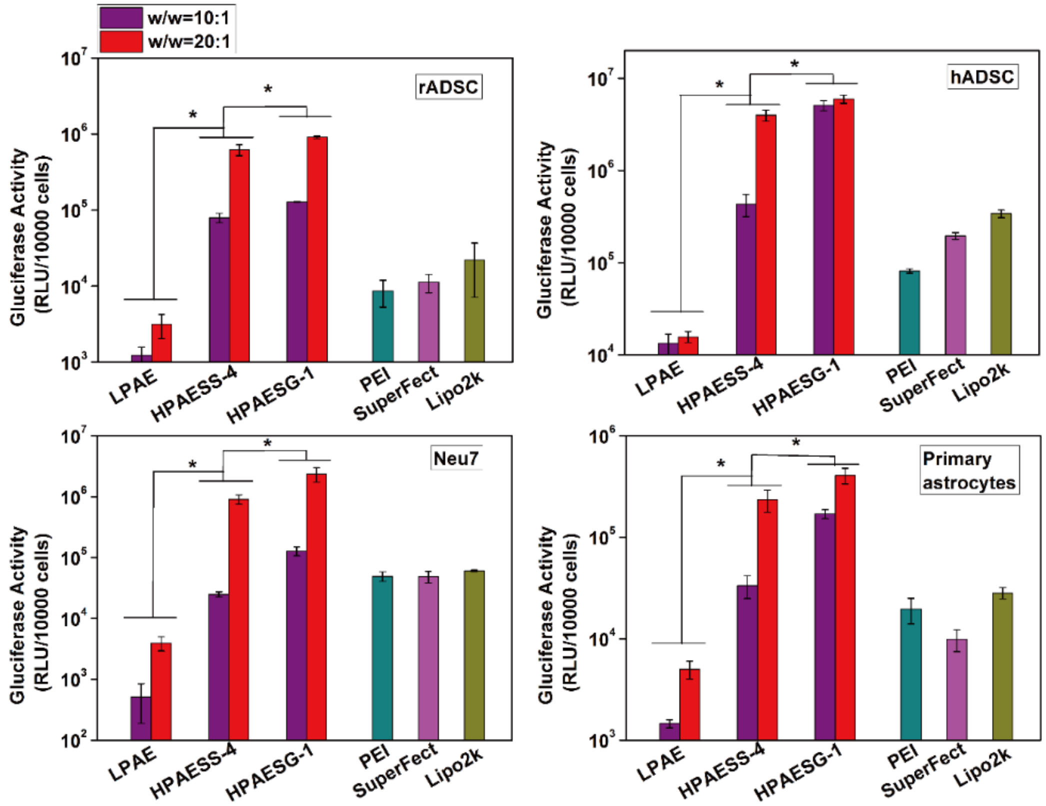

Superior transfection performance exhibited by HPAESG-1 in ADSCs and astrocytes. Gluciferase activity of cells is orders-of-magnitude higher than that of the LPAE, HPAESS-4 and commercial transfection reagents PEI, SuperFect and Lipo2k when HPAESG-1 is used at the w/w ratios of 10:1 and 20:1. Data are shown as average ± SD; n = 4. Data points marked with asterisks (*) are statistically significantly higher relative to the LPAE or HPAESS-4 group.

Supplementary Figure 22

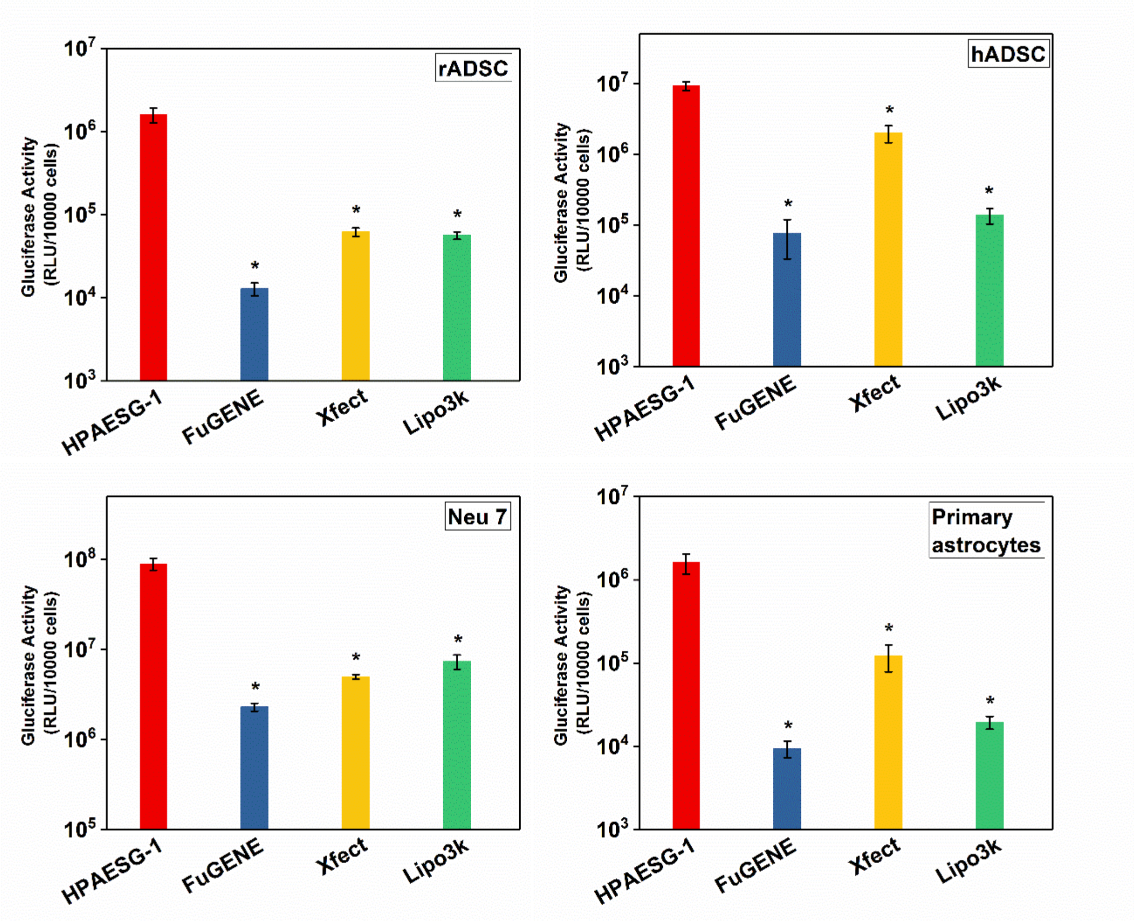

Gluciferase activity of rADSCs, hADSCs, Neu7 and primary astrocytes after transfection with HPAESG-1 (w/w = 20:1), FuGENE, Xfect and Lipo3k. Results show average of measurements conducted in quadruplicate ± SD. Data points marked with asterisks (*) are statistically significantly lower relative to the HPAESG-1 group.

Supplementary Figure 23

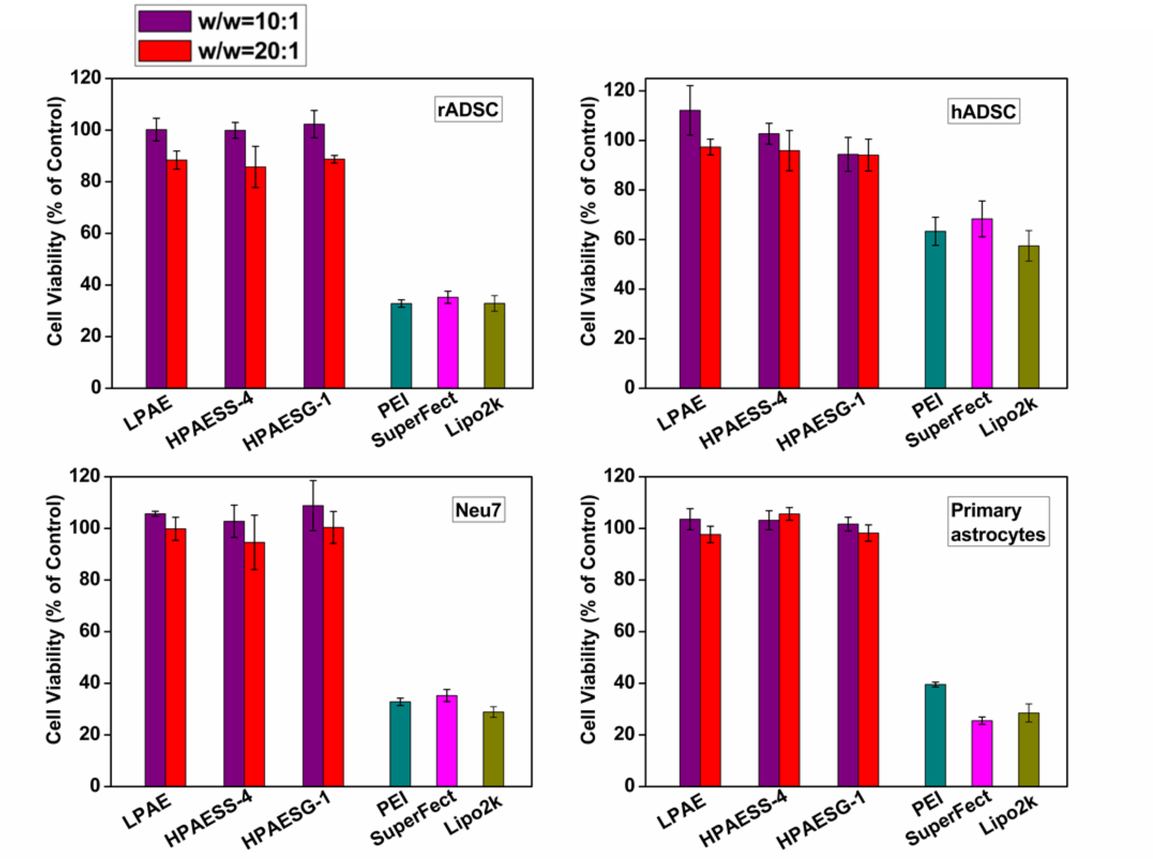

HPAESG-1 preserved high viability of ADSCs and astrocytes after transfection. Results showed average of measurements conducted in quadruplicate ± SD.

Supplementary Figure 24

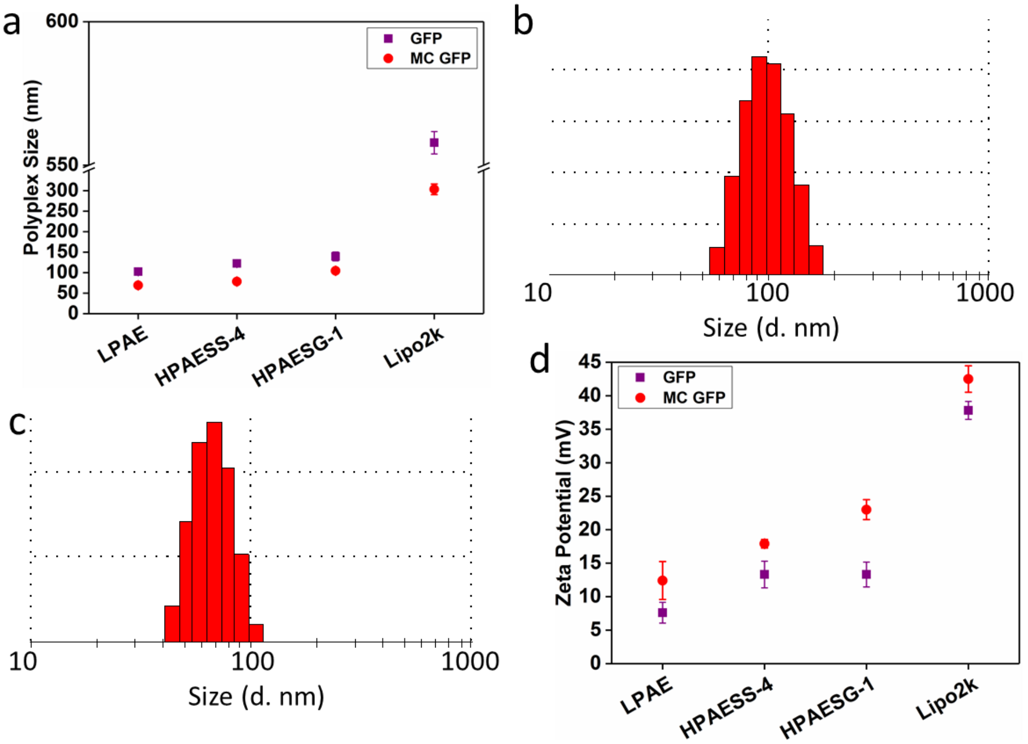

Size (a) and zeta potential (d) of the polyplexes formulated by different vectors with GFP or MC GFP at the w/w ratio of 20:1; Size distribution of HPASG-1/GF (b) and HPAESG-1/MC GFP (c) at the w/w ratio of 20:1. Data are shown as average ± SD; n = 4.

Supplementary Figure 25

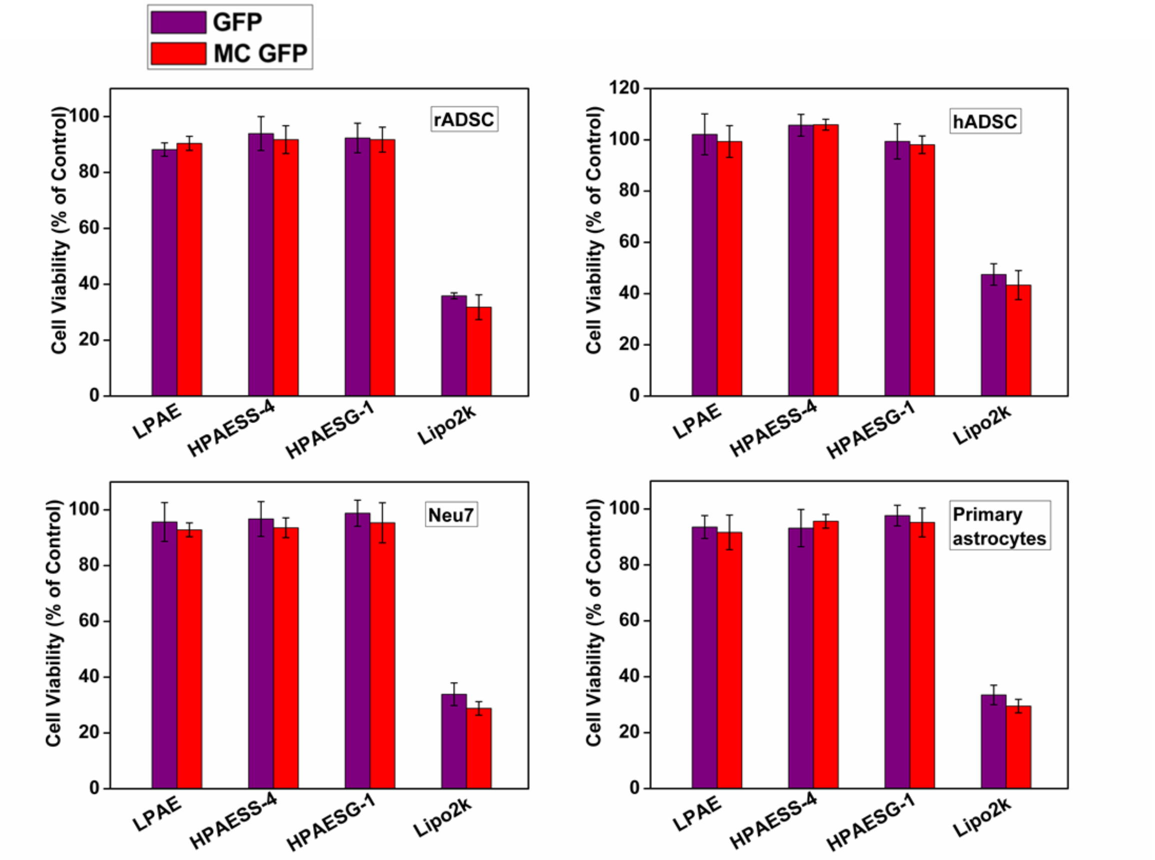

HPAESG-1/DNA and HPSESG-1/MCDNA preserved high viability of ADSCs and astrocytes after transfection at w/w =20. Results showed average of measurements conducted in quadruplicate ± SD.

Supplementary Figure 26

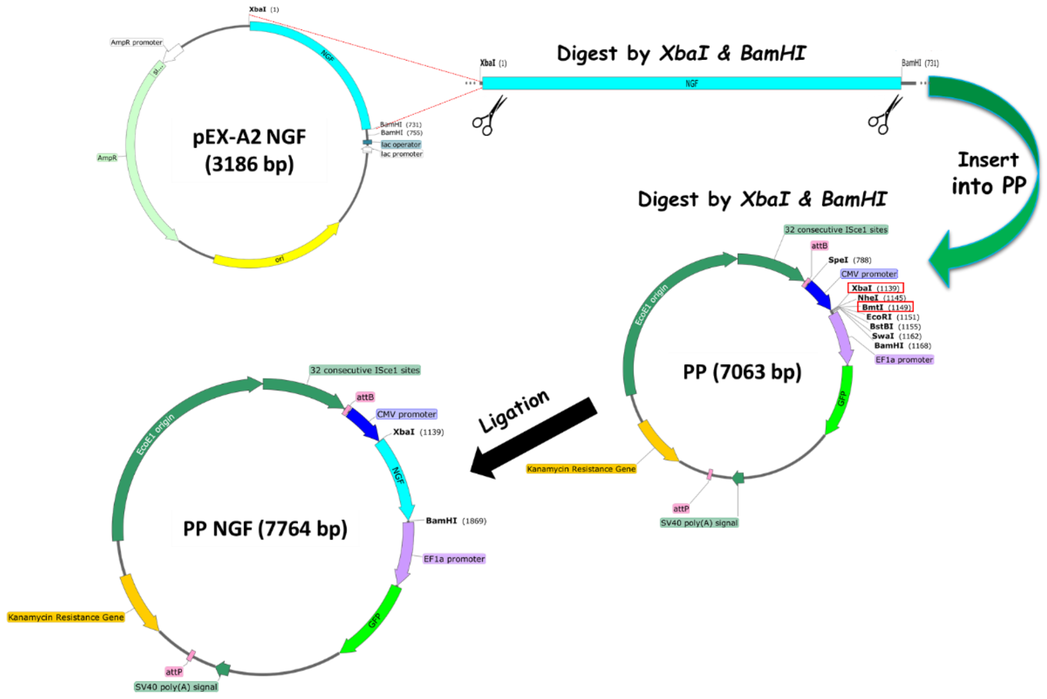

Schematic illustration of the subcloning of NGF fragment into PP MN511A1 to generate PP NGF.

NGF sequence:

TCTAGAGATGTCCATGTTGTTCTACACTCTGATCACAGCTTTTCTGATCGGCATACAGGCGGAACCACACTCA
GAGAGCAATGTCCCTGCAGGACACACCATCCCCCAAGCCCACTGGACTAAACTTCAGCATTCCCTTGACAC
TGCCCTTCGCAGAGCCCGCAGCGCCCCGGCAGCGGCGATAGCTGCACGCGTGGCGGGGCAGACCCGCAA
CATTACTGTGGACCCCAGGCTGTTTAAAAAGCGGCGACTCCGTTCACCCCGTGTGCTGTTTAGCACCCAGC
CTCCCCGTGAAGCTGCAGACACTCAGGATCTGGACTTCGAGGTCGGTGGTGCTGCCCCCTTCAACAGGAC
TCACAGGAGCAAGCGGTCATCATCCCATCCCATCTTCCACAGGGGCGAATTCTCGGTGTGTGACAGTGTCA
GCGTGTGGGTTGGGGATAAGACCACCGCCACAGACATCAAGGGCAAGGAGGTGATGGTGTTGGGAGAG
GTGAACATTAACAACAGTGTATTCAAACAGTACTTTTTTGAGACCAAGTGCCGGGACCCAAATCCCGTTGA
CAGCGGGTGCCGGGGCATTGACTCAAAGCACTGGAACTCATATTGTACCACGACTCACACCTTTGTCAAGG
CGCTGACCATGGATGGCAAGCAGGCTGCCTGGCGGTTTATCCGGATAGATACGGCCTGTGTGTGTGTGCTC
AGCAGGAAGGCTGTGAGAAGAGCCGGATCC

XbaI= TCTAGA

BamHI= GGATCC

Supplementary Figure 27

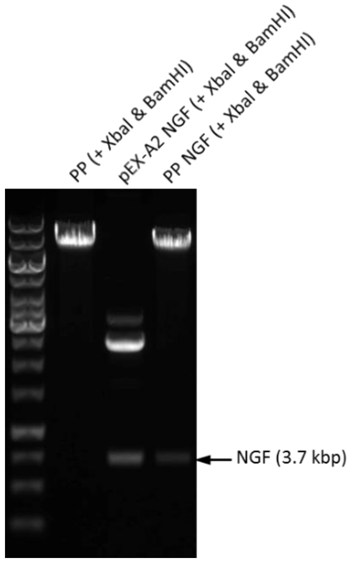

Confirmation of the structure of PP NGF by agarose gel electrophoresis.

Supplementary Figure 28

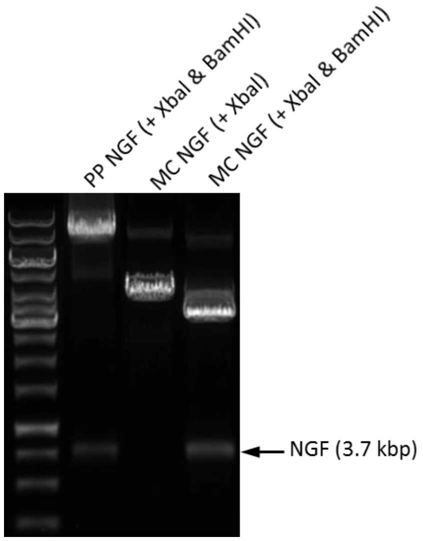

Confirmation of the structure of MC NGF by agarose gel electrophoresis.

Supplementary Figure 29

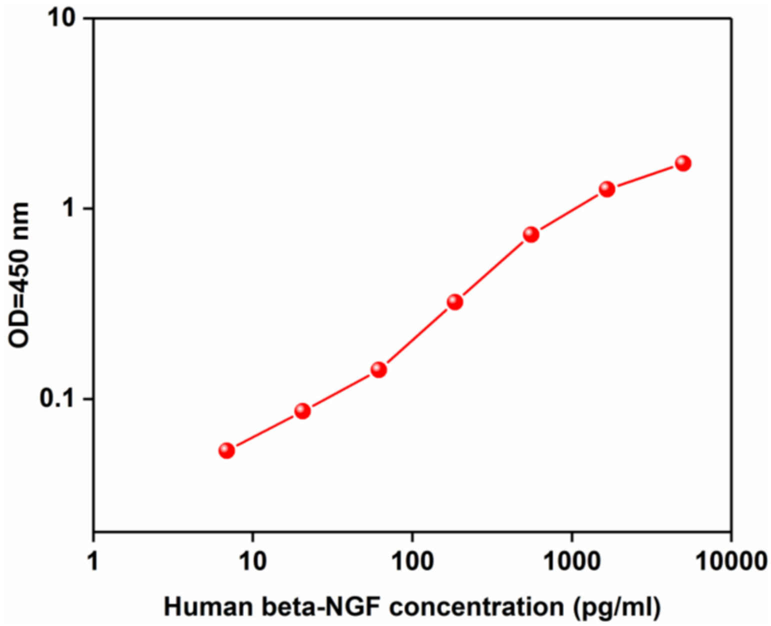

Standard curve of Human beta-NGF.

Supplementary Figure 30

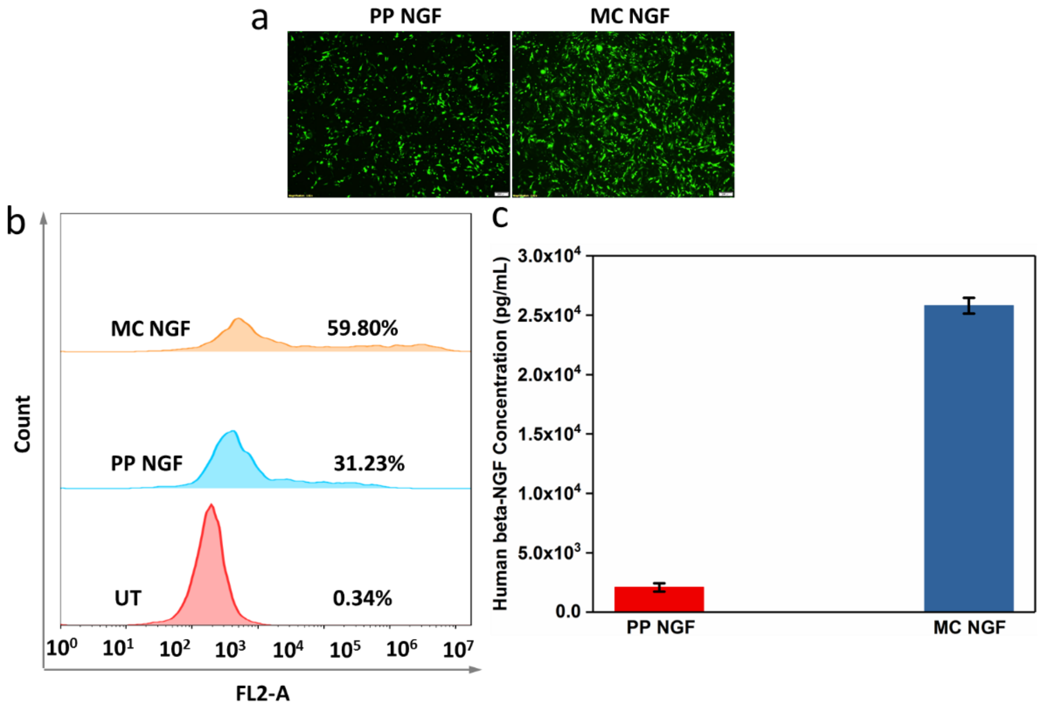

(a) GFP expression of primary astrocyte after transfection with HPAESG-1/PP NGF and HPAESG-1/MC NGF at the w/w = 20:1; (b) Representative histogram distributions of UT primary astrocyte and the ones after transfection with HPAESG-1/PP NGF and HPAESG-1/MC NGF at the w/w = 20:1; (c) Human beta-NGF concentration of supernatants from primary astrocyte astrocytes after transfection with HPAESG-1/PP NGF or HPAESG-1/MC NGF at the w/w = 20:1. Data are shown as average ± SD; n = 4.

Supplementary Figure 31

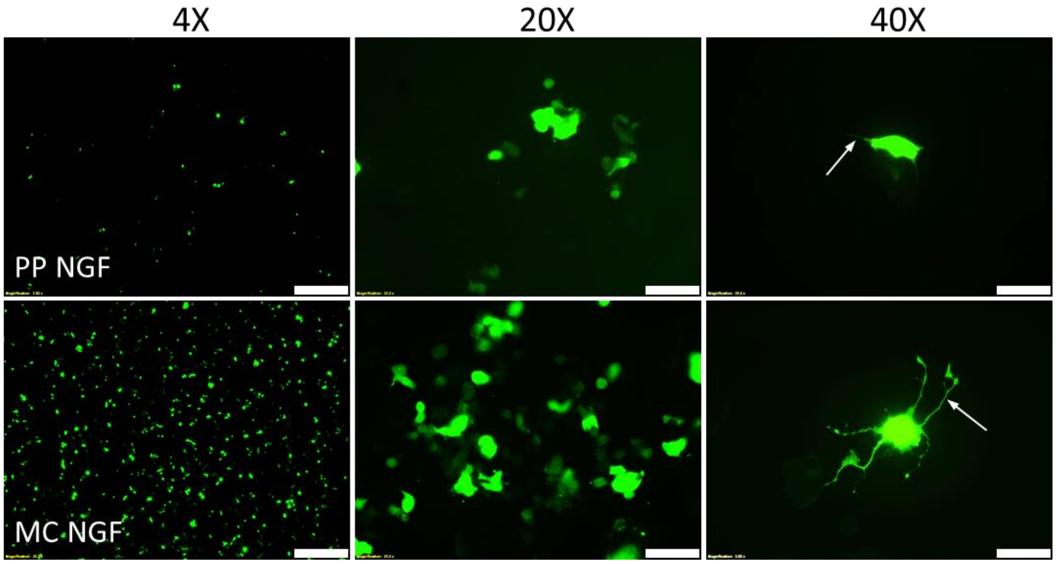

GFP images of PC12 after direct transfection with HPAESG-1/PP NGF and HPAESG-1/MC NGF at the w/w = 20:1, arrows indicate the outgrowth of neurite. The scale bars represent 500 μm.

Supplementary References

1. Zhao, T.Y. *et al.* Significance of Branching for Transfection: Synthesis of Highly Branched Degradable Functional Poly(dimethylaminoethyl methacrylate) by Vinyl Oligomer Combination. *Angew. Chem. Int. Ed.* **53**, 6095-6100 (2014).
2. Lynn, D.M. & Langer, R. Degradable poly(beta-amino esters): Synthesis, characterization, and self-assembly with plasmid DNA. *J. Am. Chem. Soc.* **122**, 10761-10768 (2000).
3. Eltoukhy, A.A., Chen, D.L., Alabi, C.A., Langer, R. & Anderson, D.G. Degradable Terpolymers with Alkyl Side Chains Demonstrate Enhanced Gene Delivery Potency and Nanoparticle Stability. *Adv. Mater.* **25**, 1487-1493 (2013).
4. Eltoukhy, A.A. *et al.* Effect of molecular weight of amine end-modified poly(β-amino ester)s on gene delivery efficiency and toxicity. *Biomaterials* **33**, 3594-3603 (2012).
5. Keeney, M. *et al.* Development of Poly(beta-amino ester)-Based Biodegradable Nanoparticles for Nonviral Delivery of Minicircle DNA. *ACS Nano* **7**, 7241-7250 (2013).
6. Zhou, D. *et al.* The transition from linear to highly branched poly(beta-amino ester)s: Branching matters for gene delivery. *Sci. Adv.* **2**, e1600102 (2016).
7. Cutlar, L. *et al.* Highly Branched Poly(beta-Amino Esters): Synthesis and Application in Gene Delivery. *Biomacromolecules* **16**, 2609-2617 (2015).
