# Supplementary material for: Highly branched poly(β-amino ester) delivery of minicircle DNA for transfection of neurodegenerative disease related cells
Source: Nat Commun. 2019 Jul 24;10:3307. doi: 10.1038/s41467-019-11190-0 (PMC6656726; doi:10.1038/s41467-019-11190-0)
Supplement: Supplementary file 2 — Reporting Summary [file 41467_2019_11190_MOESM2_ESM.pdf]

nature research

Corresponding author(s): Wenxin Wang
Last updated by author(s): Jun 14, 2019

Reporting Summary

Nature Research wishes to improve the reproducibility of the work that we publish. This form provides structure for consistency and transparency in reporting. For further information on Nature Research policies, see Authors & Referees and the Editorial Policy Checklist.

Statistics

For all statistical analyses, confirm that the following items are present in the figure legend, table legend, main text, or Methods section.

n/a	Confirmed	
☐	☒	The exact sample size (*n*) for each experimental group/condition, given as a discrete number and unit of measurement
☒	☐	A statement on whether measurements were taken from distinct samples or whether the same sample was measured repeatedly
☐	☒	The statistical test(s) used AND whether they are one- or two-sided<br>*Only common tests should be described solely by name; describe more complex techniques in the Methods section.*
☒	☐	A description of all covariates tested
☒	☐	A description of any assumptions or corrections, such as tests of normality and adjustment for multiple comparisons
☒	☐	A full description of the statistical parameters including central tendency (e.g. means) or other basic estimates (e.g. regression coefficient) AND variation (e.g. standard deviation) or associated estimates of uncertainty (e.g. confidence intervals)
☒	☐	For null hypothesis testing, the test statistic (e.g. *F*, *t*, *r*) with confidence intervals, effect sizes, degrees of freedom and *P* value noted<br>*Give P values as exact values whenever suitable.*
☒	☐	For Bayesian analysis, information on the choice of priors and Markov chain Monte Carlo settings
☒	☐	For hierarchical and complex designs, identification of the appropriate level for tests and full reporting of outcomes
☐	☒	Estimates of effect sizes (e.g. Cohen's *d*, Pearson's *r*), indicating how they were calculated

Our web collection on statistics for biologists contains articles on many of the points above.

Software and code

Policy information about availability of computer code

Data collection	NMR data were collected on 400 MHz Varian NMR spectrometer, molecular weight and dispersity of polymers were measured with Agilent 1260 GPC system.
Data analysis	NMR data were analyzed with MestReNova software. GPC data were analyzed with Agilent 1260 software.

For manuscripts utilizing custom algorithms or software that are central to the research but not yet described in published literature, software must be made available to editors/reviewers. We strongly encourage code deposition in a community repository (e.g. GitHub). See the Nature Research guidelines for submitting code & software for further information.

Data

Policy information about availability of data

All manuscripts must include a data availability statement. This statement should provide the following information, where applicable:
- Accession codes, unique identifiers, or web links for publicly available datasets
- A list of figures that have associated raw data
- A description of any restrictions on data availability

All data relevant to this study are available from the corresponding author upon reasonable request.

Field-specific reporting

Please select the one below that is the best fit for your research. If you are not sure, read the appropriate sections before making your selection.

☒ Life sciences ☐ Behavioural & social sciences ☐ Ecological, evolutionary & environmental sciences

Life sciences study design

All studies must disclose on these points even when the disclosure is negative.

Sample size	All transfection experiments were performed in quadruplicate unless otherwise stated.
Data exclusions	No data was excluded
Replication	All transfection experiments were performed in quadruplicate unless otherwise stated, with error bars indicating ±SD.
Randomization	Not involved in the study.
Blinding	Not involved in the study.

Reporting for specific materials, systems and methods

We require information from authors about some types of materials, experimental systems and methods used in many studies. Here, indicate whether each material, system or method listed is relevant to your study. If you are not sure if a list item applies to your research, read the appropriate section before selecting a response.

Materials & experimental systems

n/a	Involved in the study	
☒	☐	Antibodies
☐	☒	Eukaryotic cell lines
☒	☐	Palaeontology
☒	☐	Animals and other organisms
☒	☐	Human research participants
☒	☐	Clinical data

Methods

n/a	Involved in the study	
☒	☐	ChIP-seq
☐	☒	Flow cytometry
☒	☐	MRI-based neuroimaging

Eukaryotic cell lines

Policy information about cell lines

Cell line source(s)	HKC8, HeLa and PC12 were obtained from ATCC. hADSC was obtained from Invitrogen. Primary astrocytes were obtained from ScienCell Research Laboratory (ATCC). Neu7 was a kind gift from Prof James Fawcett. rADSC were extracted from rat adipose tissue.
Authentication	Cell lines were authenticated by the vendor by morphology check and growth curve analysis.
Mycoplasma contamination	Cell lines were routinely tested for mycoplasma comtamination.
Commonly misidentified lines (See ICLAC register)	HeLa cells were used to evaluate the gene transfection efficiency.

Flow Cytometry

Plots

Confirm that:

☒ The axis labels state the marker and fluorochrome used (e.g. CD4-FITC).

☒ The axis scales are clearly visible. Include numbers along axes only for bottom left plot of group (a 'group' is an analysis of identical markers).

☒ All plots are contour plots with outliers or pseudocolor plots.

☒ A numerical value for number of cells or percentage (with statistics) is provided.

Methodology

Sample preparation	Cells were harvested with trypsin EDTA and washed with HBSS twice and then re-suspended in PBS with 2% FBS.
Instrument	Accuri C6

Software	Accuri C6, Flowjo V10
Cell population abundance	At least 8000 cells were counted for each sample.
Gating strategy	Example of gating is provided in the Supplementary Information.

☒ Tick this box to confirm that a figure exemplifying the gating strategy is provided in the Supplementary Information.
